# The effects of poloxamer and sodium alginate mixture (Guardix-SG®) on range of motion after axillary lymph node dissection: A single-center, prospective, randomized, double-blind pilot study

**DOI:** 10.1371/journal.pone.0238284

**Published:** 2020-09-23

**Authors:** Sae Byul Lee, Sung-chan Gwark, Cheol Min Kang, Guiyun Sohn, Jisun Kim, Il Yong Chung, Jong Won Lee, Hee Jeong Kim, Beom Seok Ko, Sei-Hyun Ahn, Won Kim, Junghwa Do, Jae Yong Jeon, Jinsung Kim, Eunhae Um, Tae in Yoon, Sung-ui Jung, Minkyu Han, Byung Ho Son

**Affiliations:** 1 Division of Breast Surgery, Department of Surgery, University of Ulsan College of Medicine, Asan Medical Center, Seoul, Korea; 2 Department of Rehabilitation Medicine, University of Ulsan College of Medicine, Asan Medical Center, Seoul, Korea; 3 Division of Breast Surgery, Department of Surgery, Ulsan University Hospital, Ulsan, Korea; 4 Department of Surgery, Inje University Ilsan Paik Hospital, Goyang-si, Gyeonggi-do, Korea; 5 Division of Breast Surgery, Department of Surgery, Dongnam institute of Radiological and medical science, Busan, Korea; 6 Division of Breast Surgery, Department of Surgery, Kosin University Gospel Hospital, Busan, Korea; 7 Department of Clinical Epidemiology and Biostatistics, Asan Medical Center, Seoul, Korea; Istituto di Ricovero e Cura a Carattere Scientifico Centro di Riferimento Oncologico della Basilicata, ITALY

## Abstract

**Purpose:**

Restricted shoulder mobility is a major upper extremity dysfunction associated with lower quality of life and disability after breast cancer surgery. We hypothesized that a poloxamer and sodium alginate mixture (Guardix-SG®) applied after axillary lymph node dissection (ALND) would significantly improve shoulder range of motion (ROM) in patients with breast cancer.

**Methods:**

We conducted a double-blind, randomized, prospective study to evaluate the clinical efficacy and safety of Guardix-SG® for the prevention of upper extremity dysfunction after ALND. The primary outcome measure was shoulder ROM at baseline (T0) and 3 (T1), 6 (T2), and 12 months (T3) after surgery. Secondary outcome measures were the Disabilities of the Arm, Shoulder, and Hand score(DASH), pain associated with movement, which was assessed using a numeric rating scale, and lymphedema assessed using body composition analyzer.

**Results:**

A total of 83 women with breast cancer were randomly assigned to either the Guardix-SG® group or the control group. In the Guardix-SG® group (n = 37), Guardix-SG® was applied to the axillary region after ALND. In the control group (n = 46), ALND was performed without using Guardix-SG®.

Comparing ROM for shoulder flexion before surgery (178.2°) and 12 months after surgery (172.3°), that was restored 12 months after surgery in the Guardix-SG® group, and there was no statistically significant difference between that at before surgery and 12 months after surgery (p = 0.182). No adverse effect was observed in either group.

**Conclusions:**

The results of this study have shown that Guardix-SG® help improve shoulder ROM without causing adverse effects in patients who underwent breast cancer surgery. However, there was no statistically significant difference from the control group. A further large-scale study is needed to obtain a more conclusive conclusion.

**Trial registration:**

CRISKCT0003386; https://cris.nih.go.kr (20181207)

## Introduction

As the life expectancy of women with breast cancer is prolonged, there is a need to improve their quality of life (QOL) during or after breast cancer treatment. Adverse effects that mainly occur after breast cancer surgery include pain in the arms, sensory changes, muscle weakness, lymphedema, restricted shoulder mobility, and decreased function. Limitation of shoulder range of motion (ROM) and muscle weakness reduce activities of daily living and negatively affect QOL [1–5]. A less invasive surgical procedure and more selective treatment were used, but upper extremity dysfunction remains a serious complication after treatment [6]. The effort to reduce the complications of axillary lymph node dissection (ALND) is particularly necessary. In their prospective study, Kootstra et al. reported that the rate of complications including lymphatic edema, limitations on upper extremity mobility and shoulder ROM, and muscle weakness was significantly lower in the sentinel lymph node biopsy group than in the ALND group [7]. Patients who underwent mastectomy had more reduced shoulder ROM and a higher frequency of pectoralis muscle tightness than those who underwent breast-conserving surgery. Moreover, postoperative radiotherapy and the postoperative period are important factors. In addition, shoulder ROM was most reduced within 12 months after surgery and better thereafter [2–5]. Upper extremity functions are essential in maintaining general QOL, particularly in maintaining independent living and performing tasks that require physical strength [8]. Therefore, physical, mental, and social burdens may increase if the upper extremity functions decrease. Chronic upper extremity dysfunction is a long-term complication that occurs after breast cancer surgery, which considerably affects QOL and the performance of routine activities [9–11].

Adhesion at the surgical site after ALND is major cause of decreased shoulder ROM, pectoralis tightness, and pain. Therefore, we expect a significant reduction in these symptoms through effective anti-adhesion methods, which include minimization of intraoperative resection, use of anti-inflammatory drugs, and stimulation of plasminogen activator to prevent fibrin formation.

In recent years, methods that use physical barriers have been employed [12, 13].Materials used for the physical barriers materials include oxidized regenerated cellulose from natural polysaccharides, sodium carboxymethyl cellulose, dextran, and sodium hyaluronate. Further, synthetic polymers such as polyethylene glycoland poloxamer are used.

Guardix-SG® (HanmiPharmaceutical Co. Ltd., Seoul, Korea) is an anti-adhesion agent consisting of poloxamer and sodium alginate. It prevents adhesion by forming a physical barrier on the surface of the wound tissue [14].

In this pilot study, patients undergoing modified radical mastectomy or breast-conserving surgery were followed up for 12 months after ALND surgery. The purpose of this study was to evaluate the effect of Guardix-SG® on shoulder ROM and its safety after breast cancer surgery.

## Methods

### Participants

Between January 2016 and December 2016, a total of 83 women with breast cancer underwent ALND at Asan Medical Center. Patients with carcinoma in situ, stage IV breast cancer, other preoperative shoulder diseases and shoulder disabilities, and previous history of ipsilateral breast surgery or axillary surgery; those who underwent mastectomy with reconstruction; and those who were pregnant or lactating were excluded. The protocol used in this study was approved by the Asan Medical Center institutional review board on March 14, 2014, and all participants provided written informed consent (20140234). Patients were enrolled between January 4, 2016 and December 19, 2016, and their last follow-up date was December 19, 2017. Since registration began, enrollment was delayed due to the plan, which seems to have recently reduced the number of ALND performed after preoperative chemotherapy. The authors confirm that all ongoing and related trials for this intervention are registered.

### Interventions

This was a prospective, randomized, and double-blind pilot study. During surgery, the patients were randomly assigned to either the Guardix-SG® group or the control group using the covered paper technique. Randomization to two treatment arms was performed at a 1:1 ratio through stratified block randomization. Stratified randomization was applied to each type of surgery (mastectomy vs. breast-conserving surgery) because the effect of shoulder disability on different surgical methods may differ. Since the block random size was 10, there was some imbalance in stratification for the surgical method. Papers were numbered and covered until the intervention was assigned, and the covered papers were peeled off after completion of ALND. We applied Guardix-SG® after ALND to the axillary surface of patients in the Guardix-SG® group, whereas Guardix-SG® was not applied after surgery in the control group. All patients in both groups took part in a regular exercise programs as part of routine care, which consisted of neck rotation, neck muscle stretches, pectoralis stretches, overhead shoulder stretches, and arm circles. All patients received useful information about self-care, including general shoulder ROM exercises after surgery. All patients in both groups visited the outpatient rehabilitation department after surgery, and physiotherapy was performed, if it was necessary.

### Outcome measures

All data were prospectively collected by the double-blind method at baseline (T0) and at 3 (T1), 6 (T2), and 12 months (T3) after surgery. The primary outcome was shoulder ROM at 12 months after surgery in the affected upper extremity. Shoulder ROM has three components: abduction, flexion and horizontal abduction. All ROMs were measured in the subjects in a seated position using a goniometer based on our clinical experience. Secondary outcome measures were (1) pain associated with movement, which was assessed using a numeric rating scale (NRS) (0 represents “no pain” and 100 represents “worse pain imaginable”) and (2) lymphedema assessed using body composition analyzer (Inbody S10;Bio-sapce, Korea). We obtained values for extracellular fluid (ECF) using bioelectrical impedance spectroscopy (BIS), specific to ECF and more sensitive to localized lymphedema. The ECF ratios of the affected to unaffected arm were calculated[15]. Also, another secondary outcome was (3) Disabilities of the Arm, Shoulder, and Hand (DASH) score [16, 17]. The validated Korean version of the DASH questionnaire was used to measure symptoms and functional status based on physical function according to the severity of the upper extremity dysfunction.

### Postoperative safety assessment

Complications and drainage duration after surgery were recorded for each patient, and adverse effects were monitored throughout the study. Drainage was assessed daily starting from the day of surgery, and drainage was removed when the drainage volume decreased to less than 20 mL over a 24-h period. The overall drainage output and incidence of symptomatic seroma formation were also measured. A symptomatic seroma, defined as a palpable accumulation of fluid under the wound with symptoms, was treated by aspiration or drainage insertion. White blood cell and neutrophil counts were determined when an adverse reaction such as inflammation was suspected.

### Statistical analysis

We estimated that a sample size of 96 per group was needed to achieve 90% statistical power to detect a mean difference in shoulder ROM of 10° using a one-sided, with a standard deviation (SD) of 10° between treatment groups at a statistical significance level of 0.0083 [3]. Baseline demographic and clinical characteristics of patients in the Guardix-SG® group and the control groups were compared using the Student’s *t*-test and chi-square test. Variables with small cell sizes were analyzed using Fisher's exact test.

The shoulder ROM, pain associated with movement, lymphatic edema assessed using body composition analysis, and DASH score were included in the analysis, which was performed using a repeated-measures. Means were modeled as a function of group assignment and study visit (at baseline and at 3, 6, and 12 months). We used the Wilcoxon signed-rank test to compare the difference among each time (0, 3, 6, 12 months). The differences at each time point were compared. We show averaged lines and those values are derived from the nonparametric estimation of mean functions called local polynomial regression fitting. Data management and statistical analyses were performed using SPSS version 21.0 (SPSS, Chicago, IL, USA). The statistical methods used to estimate the 95% confidence interval.

### Ethical approval

All procedures performed in studies involving human participants were in accordance with the ethical standards of the institutional and/or national research committee and with the 1964 Helsinki declaration and its later amendments or comparable ethical standards.

## Results

### Patient characteristics

Of the 131 recruited patients, 83 were included in the study for baseline evaluation, and 48 were excluded because they did not undergo ALND. Participants were randomly assigned to either the Guardix-SG® group (n = 37) or the control group (n = 46) after primary baseline assessment. The average age was 52.0 years (SD 9.8 years) in the Guardix-SG® group and 51.3 years (SD 10.0 years) in the control group. In the Guardix-SG® group and the control group, the average tumor size was 2.6 cm (SD 2.4 cm) and 2.5 cm (SD 2.4 cm), and the mean number of lymph nodes was 2.6 (SD 9.4) and 3.9 (SD 10.6), respectively. There was no statistical difference of these between the two groups. Other general characteristics are presented in Table 1.

**Table 1:** Baseline characteristics of the subjects.

Characteristic	Guardix-SG group (N = 37)	Control group (N = 46)	*p*-value
Age (years)	52.0 (9.8)	51.3 (10.0)	0.764
BMI (kg/㎡)	25.1 (3.6)	25.5 (4.7)	0.618
Tumor size (cm)	2.6 (2.4)	2.5 (2.4)	0.775
Resected number of LN	11.9 (8.9)	15.5 (12.3)	0.134
Total drainage (mL)	422.2 (245.1)	521.5 (496.5)	0.239
Site			
Left	18 (48.6%)	27 (58.7%)	0.361
Right	19 (51.4%)	19 (41.3%)	
Surgery			
Mastectomy	24 (64.9%)	24 (52.2%)	0.381
BCS	13 (35.1%)	21 (45.6%)	
ALND only	0 (0)	1 (2.2%)	
ALND level			
Level I	13 (35.1%)	12 (26.1%)	0.116
Level II or III	23 (62.2%)	34 (73.9%)	
Unknown	1 (2.7%)	0 (0)	
T stage			
1,2	31 (83.8%)	36 (78.3%)	0.663
3,4	6 (16.2%)	10 (21.7%)	
N stage			
0,1	26 (70.3%)	32 (69.6%)	0.945
2,3	11 (29.7%)	14 (30.4%)	
Stage			
1,2	21 (58.3%)	27 (62.8%)	0.686
3	15 (41.7%)	16 (37.2%)	
Radiotherapy			
No	4 (10.8%)	7 (15.2%)	0.374
Yes	33 (89.2%)	39(84.8%)	

BCS = breast conserving surgery; BMI = body mass index; LN = lymph node; ALND = axillary lymph node dissection

**Fig 1:**
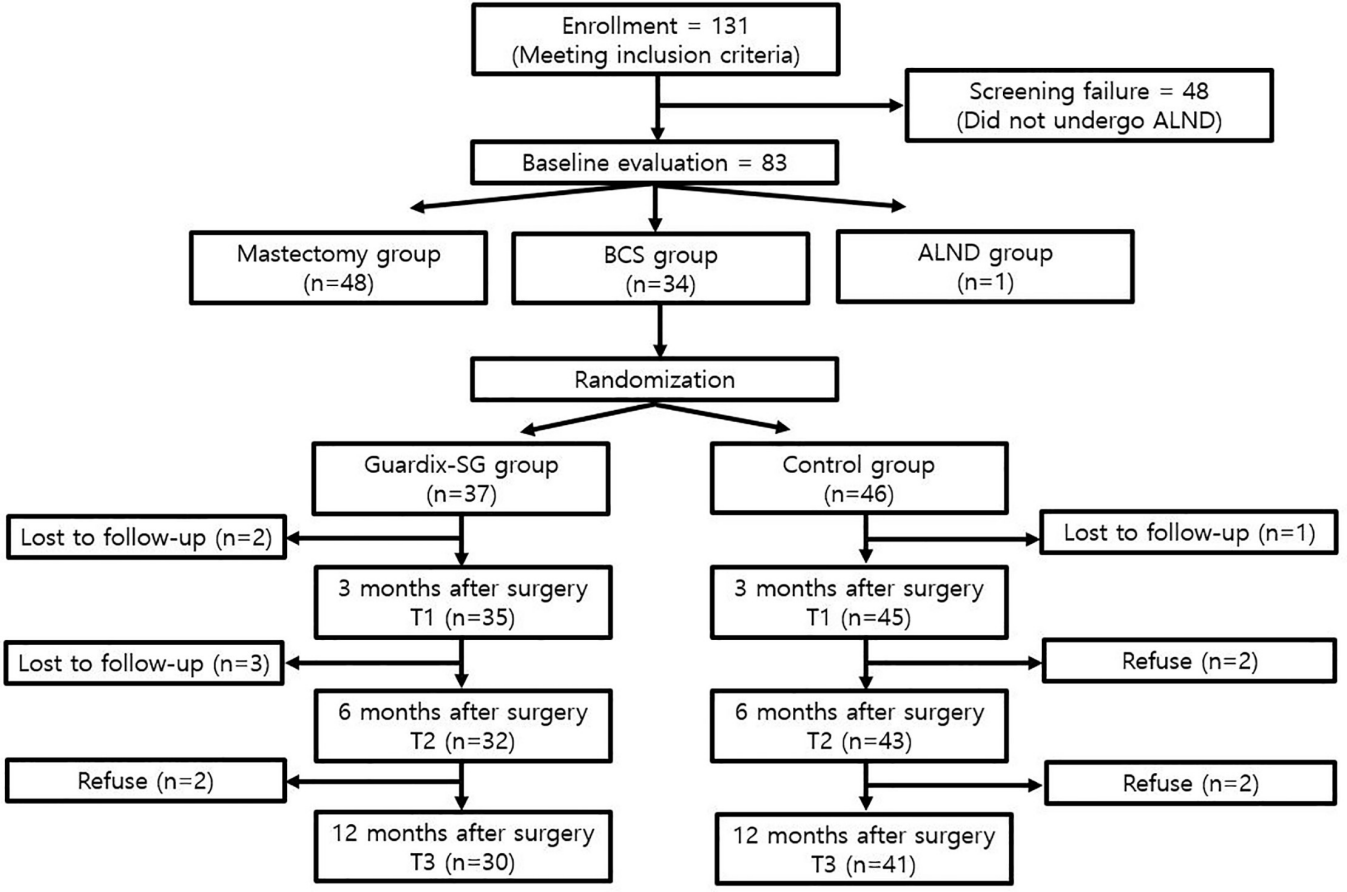
Flowchart of recruitment, randomization, and follow-up of study participants.

Follow-up was performed at 3, 6, and 12 months. In the Guardix-SG® group, 2 and 3 patients were lost during the follow-up at 3 and 6 months, respectively, and 2 refused further follow-up at 12 months; hence, the follow-up in only 30 patients was performed up to 12 months. Moreover, in the control group, 3 patients were lost during the follow-up at 3 months, and 1 and 2 refused further follow-up at 6 and 12 months, respectively; hence, the follow-up in only 41 patients was performed up to 12 months (Fig 1).

### Shoulder ROM comparison between two groups

**Fig 2:**
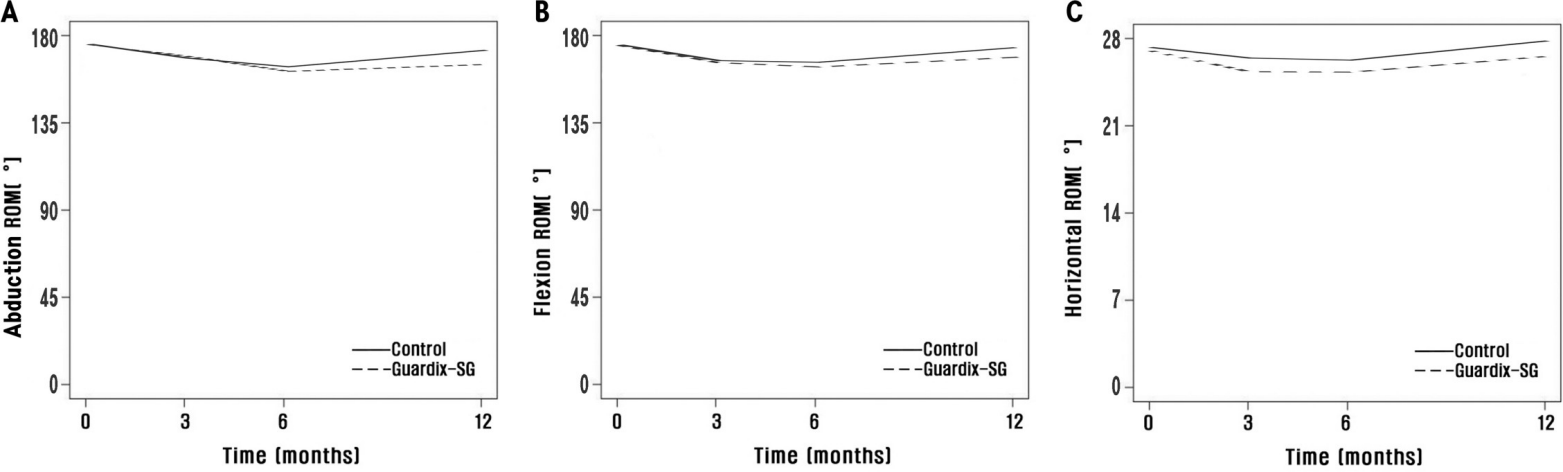
Effect of Guardix-SG® on the total shoulder range of motion (ROM). The graphs illustrate the longitudinal effect of Guardix-SG® on ROM, with the solid and dashed lines representing the Guardix-SG® group and the control group, respectively. a. Shoulder abduction. b. Shoulder flexion. c. Horizontal abduction.

At 3 months after surgery, there were no significant differences in all ROM types between both groups. And, the follow-up at 6 and 12 months indicated that all ROM types had no statistically significant in the Guardix-SG® group compared to those in the control group (Fig 2). The mean value of ROM for shoulder abduction was 164.6° and 168.1° in the Guardix-SG® group and 166.9° and 175.5° in the control group at 6 and 12 months after surgery, respectively. The mean value of ROM for shoulder flexion was 167.2° and 172.3° in the Guardix-SG® group and 169.6° and 177.2° in the control group at 6 and 12 months after surgery, respectively. The mean value of ROM for horizontal abduction was 19.3° and 22.6° in the Guardix-SG® group and 21.8° and 25.7° in the control group at 6 and 12 months after surgery, respectively, without statistical significance (Table 2).

**Table 2:** Shoulder range of movement between preoperative and postoperative (3 months, 6 months, 12 months) in each group.

	Shoulder Abduction			Shoulder Flexion			Horizontal abduction		
	mean	SD	*p*-value*	mean	SD	*p*-value*	mean	SD	*p*-value*
0 month									
Control	178.5	4.5	0.709	178.6	4.2	0.529	24.4	9.9	0.909
Guardix-SG	178.2	4.8		178.2	4.6		23.7	7.8	
3 months									
Control	171.4	18.7	0.505	170.4	14.8	0.313	22.2	11.4	0.638
Guardix-SG	172.2	13.5		169.5	11.1		19.4	9.4	
6 months									
Control	166.9	20.0	0.643	169.6	13.7	0.476	21.8	12.9	0.348
Guardix-SG	164.6	21.4		167.2	14.2		19.3	12.1	
12 months									
Control	175.5	9.6	0.918	177.2	8.1	0.516	25.7	9.4	0.330
Guardix-SG	168.1	25.8		172.3	20.3		22.6	12.6	

*student t-test

### Chronological comparison of shoulder ROM from each group

The ROM for shoulder abduction, shoulder flexion, and horizontal abduction decreased in both groups at 6 months after surgery but increased after 12 months (Fig 2). In other words, ROM, which decreased after 6 months of surgery, recovered 12 months after surgery.

Comparing ROM for shoulder flexion before surgery (178.2°) and 12 months after surgery (172.3°), that was restored 12 months after surgery in the Guardix-SG® group, and there was no statistically significant difference between that at before surgery and 12 months after surgery (*p* = 0.182). In the control group, the ROMs of shoulder flexion before surgery and 12 months after surgery were 178.6° and 177.2°, respectively. There was also no statistically significant difference (p = 0.505). And, ROM for horizontal abduction also showed similar trends (Table 3).

**Table 3:** Comparison of the change of shoulder ROM between preoperative and postoperative (3 month, 6 month, 12 month) in control and Guardix-SG^Ⓡ^-treated groups.

*p*-value	0 month		
	3m	6m	12m
Shoulder Abduction			
Total	<0.001	<0.001	0.011
Control	0.017	<0.001	0.117
Guardix-SG	0.008	0.002	0.037
Shoulder Flexion			
Total	<0.001	<0.001	0.136
Control	0.001	<0.001	0.505
Guardix-SG	<0.001	<0.001	0.182
Horizontal abduction			
Total	0.010	0.017	0.238
Control	0.206	0.319	0.069
Guardix-SG	0.014	0.022	0.854

*student t-test

### Secondary outcomes analysis

Compared to the control group, the DASH score in the Guardix-SG® group improved after 3 months of surgery, but this was not statistically significant. In the Guardix-SG® group and the control group, the median value of DASH score was 10.00 (Quartile range, 4.67–20.41) and 10.8 (Quartile range, 4.17–22.50), respectively, at 3 months of follow-up. During the follow-up, the median value of pain score was 20.00 (Quartile range, 0.00–45.00), 25.00 (Quartile range, 3.50–38.00), and 21.00 (Quartile range, 3.00–50.00) at 3, 6, and 12 months after surgery, respectively, in the control group. The median value of pain score was 11.00 (Quartile range, 2.50–33.00), 20.00 (Quartile range, 5.50–40.00), and 25.00 (Quartile range, 10.00–35.00) at 3, 6, and 12 months after surgery, respectively, in the Guardix-SG® group.

Body composition analysis showed no difference in lymphatic edema between both groups at all periods (Table 4).

**Table 4:** Pain, DASH and body composition between preoperative and postoperative (3 month, 6 month, 12 month) in control and Guardix-SG^Ⓡ^-treated groups.

	Pain			DASH			ECF ratio		
	median	Quartile range (75%)	*p*-value*	median	Quartile range (75%)	*p*-value*	median	Quartile range (75%)	*p*-value*
0 month									
Control	0	0.00–0.00	0.372	5.8	0.83–14.17	0.730	0.99	0.97–1.01	0.706
Guardix-SG	0	0.00–0.00		5.2	1.67–12.50		0.97	0.95–1.01	
3 months									
Control	20.0	0.00–45.00	0.621	10.8	4.17–22.50	0.774	1.01	0.98–1.03	0.582
Guardix-SG	11.0	2.50–33.00		10.0	4.67–20.41		1.00	0.98–1.02	
6 months									
Control	25.0	3.50–38.00	0.920	10.0	4.17–19.07	0.377	1.01	0.98–1.03	0.222
Guardix-SG	20.0	5.50–40.00		15.4	2.50–24.16		1.00	0.98–1.03	
12 months									
Control	21.0	3.00–50.00	0.679	11.2	1.67–22.50	0.513	1.00	0.97–1.03	0.090
** Guardix-SG**	**25.0**	**10.00–35.00**		**14.2**	**5.00–20.00**		**1.01**	**0.97–1.05**	

DASH = Disabilities of the Arm, Shoulder, and Hand score; ECF = extracellular fluid

*student t-test

## Discussion

Postoperative adhesions caused by wound hemorrhage, inflammation, and infection during surgery [18] are nearly the same regardless of the tissue type in the human body. In the early stage, the fibrous tissue is formed by thrombus, and the surrounding tissues attach to the wound area. The cells subsequently penetrate, multiply, and are fixed.

In a study on the effect of using Guardix-SG® in thyroid surgery, Park et al. reported that the sensory abnormality in the treatment group diminished with time, and the discomfort associated with swallowing improved owing to decreased adhesion [19]. However, there was no significant difference of pain and edema between the treatment group and the control group in this study.

Using Guardix-SG® in breast cancer surgery helped patients recover from ROM. Comparing ROM for shoulder flexion before surgery (178.2°) and 12 months after surgery (172.3°), that was restored 12 months after surgery in the Guardix-SG® group, and there was no statistically significant difference between that at before surgery and 12 months after surgery (p = 0.182).

In our study, there were no significant differences in all ROM types between both groups (Fig 2). This is because we planned to include 196 patients in the study at the onset of the study, but only 83 patients were enrolled in the study. The reason for this is that neoadjuvant therapy has recently been found to be effective in patients with positive axillary lymph nodes, resulting in a decrease in the number of patients who did not undergo ALND. Therefore, accounting for the dropout rate, the final number of patients participating in the actual study was smaller than the planned number of patients before the start of the study. In addition, after surgery, patients with symptoms or discomforts were treated with physiotherapy, which may affect the outcome. Therefore, further study to control these confounding variables is needed for more accurate results.

Guardix-SG® is known to significantly reduce postoperative adhesions [19]. Its efficacy in abdominal surgery is known to be excellent [20]. Several studies on shoulder disease have shown that Guardix-SG® reduces postoperative adhesions in animal models [21] and reduces shoulder stiffness when used as a nonsurgical treatment. After arthroscopic repair of the rotator cuff, an anti-adhesion agent was injected into the subacromial space, and the shoulder quickly recovered after surgery [21].

In our study, the mean ROM for shoulder flexion and shoulder abduction was 170°and 171°, respectively, in the control group at 3 months after surgery. The ROM for shoulder flexion reported in other studies was 143° [22], 152° [23], and 163° [24]. The mean values for shoulder flexion and abduction in healthy women were 176° and 187°, respectively [25]. Thus, the ROM for flexion and abduction in breast cancer survivors decreases by approximately 24° and 42°, respectively, compared to that in healthy women.

When muscle flexibility is lost, pain associated with exercise occurs [4]. Local or localized pain is the most frequent (20–65%) [26] adverse effect after breast cancer treatment, and it decreases QOL [27]. In addition, pain immediately after surgery can lead to chronic pain after breast cancer surgery [28]. Therefore, attempts to reduce the intensity of pain are clinically important. There is increasing evidence that pain following mastectomy is associated with fascial tissue and nerve damage [29]. According to some studies, myofascial dysfunction is common in the pectoralis major muscle, which is the result of muscle damage after transaxillary surgery [30]. According to the recommendations for clinical studies on chronic pain by the Initiative on Methods, Measurement, and Pain Assessment in Clinical Trials [31], a 1-point score change in the 10-point NRS for pain intensity does not imply a significant decrease. With respect to pain assessment, Farrar et al. [32] found that patients who rated their pain as “over-improved” and “minimal improvement” had to have at least a 1.7-point score change in pain to distinguish the pain. Thus, a reduction of less than 1.7 points in this study represents a clinically minimal change in pain [33, 34]. In this study, patients in the Guardix-SG® group had fewer disabilities than those in the control group, and the pectoralis tightness decreased, although without statistically significant difference. The pain caused by adhesion and pectoralis tightness can be alleviated or prevented through stretching exercises [24, 35, 36]. As part of routine care during the follow-up period, all patients were provided with information about self-care including shoulder and chest stretching exercises. However, the exercise program that begins during the first few days after surgery may increase seroma formation [33–36]. Previous studies have shown that short-term immobilization may be helpful but should be limited to days after surgery [37]. With Guardix-SG®, initial adhesion after surgery can be prevented during this painful period [21].

Poloxamer, which is a raw material of Guardix-SG® used in this study, exhibits a characteristic temperature-dependent sol–gel transition at a certain concentration, is biocompatible and less toxic [38], and is excreted through the kidneys [39, 40]. In contrast, alginate is a natural polysaccharide extracted from brown seaweed and is composed of ß-D-mannuronate and α-L-glucuronate. It forms a gel by cross-linking with calcium ions, has no immunological activity, and is not digested by animal cells. Calcium ions are gradually diffused out of the gel, and alginate is slowly degraded and eliminated through the urine[41]. During the study period, patients were evaluated for adverse reactions; there were no cases of adverse reaction or renal dysfunction in the treatment group and the control groups.

Through this paper, we found that Guardix-SG® improved the shoulder ROM after breast cancer surgery. It was that the recovery in the Guardix group after 12 months of surgery was greater, and, it was found that the degree of recovery recovered to almost similar level before surgery. However, there was no statistically significant difference from the control group.

This study has some limitations. First of all, we need to include more patients in the study, but we haven't. This makes the in-sample size calculation different from the primary end point of present study. In addition, the same chemotherapy and antihormonal therapy were not applied to all breast cancer patients. However, unlike previous studies that included patients with SNB surgery, this study had the advantage that there was no difference due to the surgical method since the patients who underwent only ALND surgery were included in this study. There is also a difference in the number of patients in the two groups, due to the use of a 10-size block random method, which is also a limitation of the study. In addition, the operation was performed by 8 surgeons, and the difference according to the surgical method is a limitation of this paper. In order to minimize such effects, the results were further analyzed according to ALND level, and there was no difference in the results.

## Conclusions

The results of this study have shown that Guardix-SG® help improve shoulder ROM without causing adverse effects in patients who underwent breast cancer surgery. However, there was no statistically significant difference from the control group.

## Supporting information

S1 Checklist CONSORT 2010 checklist of information to include when reporting a randomised trial*. (DOC)

S1 Data (ZIP)

S1 File (DOCX)

S2 File (DOCX)

S3 File (DOCX)

## References

[1] Hayes SC, Rye S, Battistutta D, DiSipio T, Newman B. Upper-body morbidity following breast cancer treatment is common, may persist longer-term and adversely influences quality of life. Health Qual Life Outcomes. 2010; 8: 92 10.1186/1477-7525-8-92 20804558 PMC2940926

[2] Kwan W, Jackson J, Weir LM, Dingee C, McGregor G, Olivotto IA. Chronic arm morbidity after curative breast cancer treatment: prevalence and impact on quality of life. J Clin Oncol. 2002; 20(20): 4242–4248. 10.1200/JCO.2002.09.018 12377968

[3] Yang EJ, Park WB, Seo KS, Kim SW, Heo CY, Lim JY. Longitudinal change of treatment-related upper limb dysfunction and its impact on late dysfunction in breast cancer survivors: a prospective cohort study. J Surg Oncol. 2010; 101(1): 84–91. 10.1002/jso.21435 19924721

[4] Cheville AL, Tchou J. Barriers to rehabilitation following surgery for primary breast cancer. J Surg Oncol. 2007; 95(5): 409–418. 10.1002/jso.20782 17457830

[5] Stubblefield MD, Custodio CM. Upper-extremity pain disorders in breast cancer. Arch Phys Med Rehabil. 2006; 87(3 Suppl 1): S96–99; quiz S100-101. 10.1016/j.apmr.2005.12.017 16500198

[6] Satariano WA, Ragland DR, DeLorenze GN. Limitations in upper-body strength associated with breast cancer: a comparison of black and white women. J Clin Epidemiol. 1996; 49(5): 535–544. 10.1016/0895-4356(95)00565-x 8636727

[7] Kootstra JJ, Hoekstra-Weebers JE, Rietman JS, de Vries J, Baas PC, Geertzen JH, et al A longitudinal comparison of arm morbidity in stage I-II breast cancer patients treated with sentinel lymph node biopsy, sentinel lymph node biopsy followed by completion lymph node dissection, or axillary lymph node dissection. Ann Surg Oncol. 2010; 17(9): 2384–2394. 10.1245/s10434-010-0981-8 20221902 PMC2924495

[8] Ferrell BR, Grant MM, Funk B, Otis-Green S, Garcia N. Quality of life in breast cancer survivors as identified by focus groups. Psychooncology. 1997; 6(1): 13–23. 10.1002/(sici)1099-1611(199703)6:1<13::aid-pon231>3.0.co;2-s 9126712

[9] Bruner DW, Wasserman T. The impact on quality of life by radiation late effects. Int J Radiat Oncol Biol Phys. 1995; 31(5): 1353–1355. 10.1016/0360-3016(95)00042-W 7713794

[10] Swedborg I, Wallgren A. The effect of pre- and postmastectomy radiotherapy on the degree of edema, shoulder-joint mobility, and gripping force. Cancer. 1981; 47(5): 877–881. 10.1002/1097-0142(19810301)47:5&lt;877::aid-cncr2820470511&gt;3.0.co;2-3 7013962

[11] Tobin MB, Lacey HJ, Meyer L, Mortimer PS. The psychological morbidity of breast cancer-related arm swelling. Psychological morbidity of lymphoedema. Cancer. 1993; 72(11): 3248–3252. 10.1002/1097-0142(19931201)72:11&lt;3248::aid-cncr2820721119&gt;3.0.co;2-z 8242549

[12] Falk K, Bjorquist P, Stromqvist M, Holmdahl L. Reduction of experimental adhesion formation by inhibition of plasminogen activator inhibitor type 1. Br J Surg. 2001; 88(2): 286–289. 10.1046/j.1365-2168.2001.01647.x 11167882

[13] Burns JW, Colt MJ, Burgees LS, Skinner KC. Preclinical evaluation of Seprafilm bioresorbable membrane. Eur J Surg Suppl. 1997; (577): 40–48. 9076451

[14] Arnold PB, Green CW, Foresman PA, Rodeheaver GT. Evaluation of resorbable barriers for preventing surgical adhesions. Fertil Steril. 2000; 73(1): 157–161. 10.1016/s0015-0282(99)00464-1 10632432

[15] Kim L, Jeon JY, Sung IY, Jeong SY, Do JH, Kim HJ. Prediction of treatment outcome with bioimpedance measurements in breast cancer related lymphedema patients. Ann Rehabil Med. 2011; 35(5): 687–693. 10.5535/arm.2011.35.5.687 22506192 PMC3309267

[16] Hudak PL, Amadio PC, Bombardier C, The Upper Extremity Collaborative Group (UECG). Development of an upper extremity outcome measure: the DASH (disabilities of the arm, shoulder and hand). Am J Ind Med. 1996; 29(6): 602–608. 10.1002/(sici)1097-0274(199606)29:6<602::aid-ajim4>3.0.co;2-l 8773720

[17] Lee JY, Lim JY, Oh JH, Ko YM. Cross-cultural adaptation and clinical evaluation of a Korean version of the disabilities of arm, shoulder, and hand outcome questionnaire (K-DASH). J Shoulder Elbow Surg. 2008; 17(4): 570–574. 10.1016/j.jse.2007.12.005 18472283

[18] Kimmelman CP, Edelstein DR, Cheng HJ. Sepragel sinus (hylan B) as a postsurgical dressing for endoscopic sinus surgery. Otolaryngol Head Neck Surg. 2001; 125(6): 603–608. 10.1067/mhn.2001.120229 11743460

[19] Kim JH, Lee JH, Yoon JH, Chang JH, Bae JH, Kim KS. Antiadhesive effect of the mixed solution of sodium hyaluronate and sodium carboxymethylcellulose after endoscopic sinus surgery. Am J Rhinol. 2007; 21(1): 95–99. 10.2500/ajr.2007.21.2911 17283569

[20] Yu YD, Kim DS, Jung SW, Han JH, Suh SO. Influence of anti-adhesive agent on incidence of bile leakage after liver resection: a prospective cohort study. Int J Surg. 2016; 31: 40–46. 10.1016/j.ijsu.2016.05.062 27260310

[21] Hong JH, Choe JW, Kwon GY, Cho DY, Sohn DS, Kim SW, et al The effects of barrier materials on reduction of pericardial adhesion formation in rabbits: a comparative study of a hyaluronan-based solution and a temperature sensitive poloxamer solution/gel material. J Surg Res. 2011; 166(2): 206–213. 10.1016/j.jss.2010.09.034 21035131

[22] Hayes S, Battistutta D, Newman B. Objective and subjective upper body function six months following diagnosis of breast cancer. Breast Cancer Res Treat. 2005; 94(1): 1–10. 10.1007/s10549-005-5991-z 16172793

[23] Kaya T, Karatepe AG, Gunaydn R, Yetis H, Uslu A. Disability and health-related quality of life after breast cancer surgery: relation to impairments. South Med J. 2010; 103(1): 37–41. 10.1097/SMJ.0b013e3181c38c41 19996840

[24] Cinar N, Seckin U, Keskin D, Bodur H, Bozkurt B, Cengiz O. The effectiveness of early rehabilitation in patients with modified radical mastectomy. Cancer Nurs. 2008; 31(2): 160–165. 10.1097/01.NCC.0000305696.12873.0e 18490892

[25] Barnes CJ, Van Steyn SJ, Fischer RA. The effects of age, sex, and shoulder dominance on range of motion of the shoulder. J Shoulder Elbow Surg. 2001; 10(3): 242–246. 10.1067/mse.2001.115270 11408905

[26] Randal J. Post-mastectomy pain found to be common: treatment options sparse, but growing. J Natl Cancer Inst. 1998; 90(10): 731–732. 10.1093/jnci/90.10.731 9605640

[27] Rietman JS, Dijkstra PU, Debreczeni R, Geertzen JH, Robinson DP, De Vries J. Impairments, disabilities and health related quality of life after treatment for breast cancer: a follow-up study 2.7 years after surgery. Disabil Rehabil. 2004; 26(2): 78–84. 10.1080/09638280310001629642 14668143

[28] Perkins FM, Kehlet H. Chronic pain as an outcome of surgery. A review of predictive factors. Anesthesiology. 2000; 93(4): 1123–1133. 10.1097/00000542-200010000-00038 11020770

[29] Kudel I, Edwards RR, Kozachik S, Block BM, Agarwal S, Heinberg LJ, et al Predictors and consequences of multiple persistent postmastectomy pains. J Pain Symptom Manage. 2007; 34(6): 619–627. 10.1016/j.jpainsymman.2007.01.013 17629668

[30] Cummings M. Myofascial pain from pectoralis major following trans-axillary surgery. Acupunct Med. 2003; 21(3): 105–107. 10.1136/aim.21.3.105 14620306

[31] Dworkin RH, Turk DC, Wyrwich KW, Beaton D, Cleeland CS, Farrar JT, et al Interpreting the clinical importance of treatment outcomes in chronic pain clinical trials: IMMPACT recommendations. J Pain. 2008; 9(2): 105–121. 10.1016/j.jpain.2007.09.005 18055266

[32] Farrar JT, Young JP Jr., LaMoreaux L, Werth JL, Poole RM. Clinical importance of changes in chronic pain intensity measured on an 11-point numerical pain rating scale. Pain. 2001; 94(2): 149–158. 10.1016/s0304-3959(01)00349-9 11690728

[33] Bird SB, Dickson EW. Clinically significant changes in pain along the visual analog scale. Ann Emerg Med. 2001; 38(6): 639–643. 10.1067/mem.2001.118012 11719742

[34] Cepeda MS, Africano JM, Polo R, Alcala R, Carr DB. What decline in pain intensity is meaningful to patients with acute pain? Pain. 2003; 105(1–2): 151–157. 10.1016/s0304-3959(03)00176-3 14499431

[35] Box RC, Reul-Hirche HM, Bullock-Saxton JE, Furnival CM. Shoulder movement after breast cancer surgery: results of a randomised controlled study of postoperative physiotherapy. Breast Cancer Res Treat. 2002; 75(1): 35–50. 10.1023/a:1016571204924 12500933

[36] Morimoto T, Tamura A, Ichihara T, Minakawa T, Kuwamura Y, Miki Y, et al Evaluation of a new rehabilitation program for postoperative patients with breast cancer. Nurs Health Sci. 2003; 5(4): 275–282. 10.1046/j.1442-2018.2003.00163.x 14622379

[37] Jarvinen TA, Jarvinen TL, Kaariainen M, Kalimo H, Jarvinen M. Muscle injuries: biology and treatment. Am J Sports Med. 2005; 33(5): 745–764. 10.1177/0363546505274714 15851777

[38] Oh SH, Kim JK, Song KS, Noh SM, Ghil SH, Yuk SH, et al Prevention of postsurgical tissue adhesion by anti-inflammatory drug-loaded pluronic mixtures with sol-gel transition behavior. J Biomed Mater Res A. 2005; 72(3): 306–316. 10.1002/jbm.a.30239 15654699

[39] Grindel JM, Jaworski T, Emanuele RM, Culbreth P. Pharmacokinetics of a novel surface-active agent, purified poloxamer 188, in rat, rabbit, dog and man. Biopharm Drug Dispos. 2002; 23(3): 87–103. 10.1002/bdd.297 12173548

[40] Li C, Palmer WK, Johnston TP. Disposition of poloxamer 407 in rats following a single intraperitoneal injection assessed using a simplified colorimetric assay. J Pharm Biomed Anal. 1996; 14(5): 659–665. 10.1016/0731-7085(95)01621-x 8738197

[41] Kataoka K, Suzuki Y, Kitada M, Ohnishi K, Suzuki K, Tanihara M, et al Alginate, a bioresorbable material derived from brown seaweed, enhances elongation of amputated axons of spinal cord in infant rats. J Biomed Mater Res. 2001; 54(3): 373–384. 10.1002/1097-4636(20010305)54:3&lt;373::aid-jbm90&gt;3.0.co;2-q 11189043

