# Supplementary material for: The effects of poloxamer and sodium alginate mixture (Guardix-SG®) on range of motion after axillary lymph node dissection: A single-center, prospective, randomized, double-blind pilot study
Source: PLoS One. 2020 Sep 23;15(9):e0238284. doi: 10.1371/journal.pone.0238284 (PMC7510996; doi:10.1371/journal.pone.0238284)
Supplement: S2 File — (DOCX) [file pone.0238284.s004.docx]

**임상시험계획서**

유착방지제(상품명 : GUARDIX-SG^Ⓡ^)

**제목 :** 유방암으로 변형 근치 유방절제술 혹은 유방보존수술 및 액와부 림프절 곽청술을 시행 받을 환자에서 Poloxamer 와 Alginate 혼합용액(Guardix-SG) 도포가 어깨 관절 가동범위에 미치는 영향: 단일기관, 전향적, 무작위배정, 비활성 대조군, 우월성, 이중맹검 연구자 임상시험

Ver 5.0

Revised Date : 2015.09.17

**CONFIDENTIAL**

본 임상시험 계획서에 포함된 정보는 시험기관장, 임상시험 책임자, 임상시험 담당자, 관련 행정기관, 임상시험기관의 IRB를 위한 것입니다. 본 계획서는 임상시험을 실시 또는 평가하기 위한 목적으로만 사용할 수 있으며, 임상시험에 관련되지 않은 자에게 공개되어서는 안되며, 문서의 기밀사항을 보장해 주시기 바랍니다.

**목 차**

| 연구일정표 ---------------------------------------------------------------------------------- | | 3 |
| --- | --- | --- |
| 1. | 임상시험의 명칭 및 단계 ------------------------------------------------------- | 4 |
| 2. | 임상시험 실시기관 및 주소 ---------------------------------------------------- | 5 |
| 3. | 임상시험책임자, 담당자, 공동연구자의 성명 및 직명 -------------------------- | 5 |
| 4. | 임상시험용 의료기기 관리자의 성명 및 직명 ---------------------------------- | 5 |
| 5. | 임상시험 의뢰자명 및 주소 ----------------------------------------------------- | 5 |
| 6. | 임상시험의 배경 및 목적 -------------------------------------------------------- | 6 |
| 7. | 임상시험용 의료기기의 사용목적 ----------------------------------------------- | 8 |
| 8. | 피험자 선정기준, 제외기준, 목표한 피험자 수 및 그 근거--------------------- | 9 |
| 9. | 임상시험기간 -------------------------------------------------------------------- | 13 |
| 10. | 임상시험방법 --------------------------------------------------------------------- | 14 |
| 11. | 관찰항목 ∙ 임상검사항목 및 관찰검사 방법 ------------------------------------ | 17 |
| 12. | 예측 부작용 및 사용상의 주의사항 --------------------------------------------- | 27 |
| 13. | 중지 및 중도탈락 기준 ---------------------------------------------------------- | 29 |
| 14. | 성능의 평가기준 ----------------------------------------------------------------- | 31 |
| 15. | 유효성의 평가기준, 평가방법, 해석방법(통계분석방법) ------------------------ | 32 |
| 16. | 이상반응을 포함한 안전성의 평가기준, 평가방법 및 보고방법 -------------- | 35 |
| 17. | 그 밖에 임상 시험을 안전하고 과학적으로 실시하기 위하여 필요한 사항 -- | 42 |
| 18. | 피험자 보상에 대한 규약 -------------------------------------------------------- | 43 |
| 19. | 임상시험 후 피험자의 진료에 관한 사항 --------------------------------------- | 45 |
| 20. | 피험자의 안전보호에 관한 대책 ------------------------------------------------ | 46 |
| 21. | 참고문헌 -------------------------------------------------------------------------- | 48 |

**연구일정표**

| 구분 | 처치 및 관찰기간 | | | | | |
| --- | --- | --- | --- | --- | --- | --- |
| 방문수 | Visit 1 | Visit 2^1^ | | Visit 3 | Visit 4 | Visit 5 |
|  | 수술전 | 수술  직후 | 수술후  7일 | 수술후  3개월 | 수술후  6개월 | 수술후  12개월 |
| Visit Window (일) |  |  | ± 2 | ± 14 | ± 14 | ± 30 |
| 선정/제외 기준 | ○ |  |  |  |  |  |
| 서면 동의서 | ○ |  |  |  |  |  |
| 기초정보 및 의학적 병력 | ○ |  |  |  |  |  |
| 혈액 검사^2^ | ○ |  | ○ |  |  |  |
| 배액량 측정³ |  | ○ | ○ |  |  |  |
| 체성분분석 | ○ |  |  | ○ | ○ | ○ |
| 시험의료기기 처방 |  | ○ |  |  |  |  |
| 어깨관절가동범위 측정 | ○ |  |  | ○ | ○ | ○ |
| 상지운동 수행능력 평가 | ○ |  |  | ○ | ○ | ○ |
| 통증평가 | ○ |  |  | ○ | ○ | ○ |
| 이상반응평가 |  |  | ○ | ○ | ○ | ○ |

1. visit2에 해당되는 혈액검사 및 이상반응 평가는 퇴원일에 이상반응 평가를 진행하거나 수술 후 7일에 재방문 하여 평가할 수 있다.

2. 혈액 검사 - WBC, Neutrophil

수술 후 7일에 시행 예정인 혈액검사는 수술 후 발열이나 수술부위의 발적 등 이상반응이 있는 경우에만 백혈구 및 염증 수치 증가여부를 확인하기 위해 CBC, CRP 등의 검사를 시행하며 수술 후 7일 이내의 검사결과를 사용할 수 있다.

3. 배액량측정 - 수술 후 7일까지 2일 간격으로 하고 퇴원 시 마지막 측정을 하고 이후에 종료한다.

**1. 임상시험의 명칭**

임상시험 명칭

: 유방암으로 변형 근치 유방절제술 혹은 유방보존수술 및 액와부림프절곽청술을 시행 받을 환자에서 Poloxamer 와 Alginate 혼합용액(Guardix-SG) 도포가 어깨 관절 가동범위에 미치는 영향: 단일기관, 전향적, 무작위배정, 비활성 대조군, 우월성, 이중맹검 연구자 임상시험

**2. 임상시험 실시기관 명 및 소재지**

|  | 실시기관명 | 주 소 | 대표번호 |
| --- | --- | --- | --- |
| 1 | 서울아산병원 | 서울시 송파구 올림픽로43길 88 | 1688-7575 |

**3. 임상시험 책임자, 담당자, 공동 연구자의 성명 및 직명**

[서울아산병원]

| 소속기관명 | 당담업무 | 소속과 | 직 명 | 성 명 |
| --- | --- | --- | --- | --- |
| 서울아산병원 | 책임자 | 유방내분비외과 | 교 수 | 손 병 호 |
| 서울아산병원 | 공동연구자 | 유방내분비외과 | 교 수 | 안 세 현 |
| 서울아산병원 | 공동연구자 | 재활의학과 | 부 교 수 | 전 재 용 |
| 서울아산병원 | 공동연구자 | 유방내분비외과 | 부 교 수 | 이 종 원 |
| 서울아산병원 | 공동연구자 | 유방내분비외과 | 임 상 부 교 수 | 고 범 석 |
| 서울아산병원 | 공동연구자 | 유방내분비외과 | 임 상 조 교 수 | 김 희 정 |
| 서울아산병원 | 공동연구자 | 유방내분비외과 | 임 상 전 임 강 사 | 김 지 선 |
| 서울아산병원 | 공동연구자 | 유방내분비외과 | 임 상 강 사 | 이 새 별 |
| 서울아산병원 | 공동연구자 | 재활의학과 | 조 교 수 | 김 원 |

**4. 임상시험용 의료기기 관리자의 성명 및 직명**

| 소속기관명 | 직 명 | 성 명 |
| --- | --- | --- |
| 서울아산병원 | 교 수 | 손 병 호 |
| 서울아산병원 | 임 상 강 사 | 이 새 별 |

의료기기관리자 선정에 대한 사유

본 임상시험용 의료기기는 실온에서 보관가능하며 별도의 보관 장치를 필요로 하지 않는 의료기기이다. 또한 피험자의 수술 시 바로 적용하는 것으로 임상시험약국에 보관하는 것 보다는 연구자가 의료기기를 관리하는 것이 연구의 진행에 적합할 것으로 판단된다.

**5. 임상시험 의뢰자명 및 주소**

**5-1. 의뢰자**

서울아산병원 유방 내분비외과 손병호 교수

**5-2. 의뢰처 주소**

서울특별시 송파구 올림픽로43길 88

**5-3. 연락처**

1688-7575

**6. 임상시험의 목적 및 배경**

**6-1. 임상시험의 목적**

유방암으로 변형 근치 유방 절제술 혹은 유방보존수술 및 액와부림프절곽청술을 시행 받을 환자에서 수술부위에 Poloxamer 와 Alginate 혼합용액(Guardix SG®)을 도포하는 시험군과 도포하지 않는 대조군 간의 림프절곽청술의 주요 합병증 중 하나인 어깨 관절 가동범위의 차이를 알아보고 합병증을 줄일 수 있는지 보고하고자 한다.

**6-2. 임상시험의 배경**

유방암 수술 후 팔의 통증, 감각변화, 근력저하, 림프부종, 어깨관절, 기능저하 등 다양한 상지부작용이 발생한다. 어깨관절 가동범위의 제한과 근력약화 등은 일상생활 활동을 감소시키고 삶의 질에 부정적인 영향을 미친다[2-6]. 특히 액와부림프절곽청술을 시행하는 경우 나타날 수 있는 합병증을 줄이기 위한 치료 및 노력이 필요하다. Kootstra등은 전향적 연구를 통해 상지운동, 근력, 어깨관절가동범위, 림프 부종 등의 합병증이 액와부림프절곽청술을 시행한 군에 비해 감시림프절 발생군에서 통계적으로 유의하게 낮음을 보고하였다[1].

어깨관절 가동범위 감소 빈도와 관련된 유의한 인자는 치료방법과 수술 후 경과기간이 영향을 미친다. 유방절제술을 시행 받은 환자가 유방보존술을 시행 받은 환자에 비해 어깨 관절 운동범위 감소 및 pectoralis tightness 빈도가 더 높으며, 수술 후 방사선 치료도 중요한 요소다. 또한 수술 후 12개월 이내에 어깨관절 운동범위가 감소 빈도가 가장 크며 이후 감소한다[3-6].

유방전절제술 후 수술부위 유착은 어깨관절 운동범위 감소, pectoralis tightness, 통증 등의 중요한 유발 인자로 수술 시 효과적인 유착방지 방법을 통해 상기 증상의 유의한 감소를 기대한다. 기존에 선행되었던 HA-CMC 연구는 유방전절제술 후 전거근(serratus anterior muscle)과 대흉근의 표면에 HA-CMC을 적용하여 유착을 감소시켜 유방암 환자의 어깨 관절 가동범위(ROM)향상 및 Pain 완화를 확인할 수 있었다[17]. 여타의 연구 또한 HA-CMC를 적용하여 유착을 감소시켜 관절가동범위의 향상을 확인할 수 있었다[21].

유착을 방지하는 방법으로 알려진 것은 수술 시 창상을 최소화하거나, 항염제의 사용 혹은 섬유소의 형성을 막기 위한 tissue plasminogen activator의 활성화 방법 등이 있으며[18], 최근에는 물리적 장벽(barrier)을 사용하는 방법이 개발되어 사용되고 있다[19]. Barrier의 이용은 조직의 상처가 치유되는 동안 물리적인 장벽으로 작용하여, 인접한 조직 사이에 유착이 형성되는 것을 막아줄 수 있다. 또한 Barrier는 일정기간의 상처치유가 끝난 후에는 분해 혹은 흡수되어 제거되어야 하며, Barrier용 재료자체 혹은 그 분해산물이 인체에 무해하여야 한다. 이러한 유착방지용 barrier에 사용되는 재료로는 천연에서 유래한 다당류로부터 만들어진 oxidized regenerated cellulose, sodium carboxymethyl cellulose (CMC), dextran, sodium hyaluronate (HA) 등이 있으며, 합성고분자로는 polyethylene glycol (PEG), Poloxamer, Goretex 등이 있다.

물리적 장벽역할을 하는 유착방지제는 다양한 재료로부터 만들어지며 그 형태도 다양하다. 이중 액체형태의 유착 방지제는 창상아래에 직접 도포함으로써 사용이 간편하고, 복강경 수술에서도 사용될 수 있어 많이 사용되지만 용액으로서 유동성이 있어 흘러내릴 경우 그 효과가 감소되는 단점이 있다.

GUARDIX-SG^Ⓡ^는 이러한 단점을 극복한 새로운 형태의 유착방지로서 합성 고분자인 Poloxamer와 천연 고분자인 Alginate로 제조되어 실온에서는 액체 상태를 유지하고 있으나 수술 부위에 적용하면 체온에 반응하여 즉시 젤 형태로 작용하는 온도 감응성 유착방지제로 생체 내 분해 실험에서 7일까지 재료가 남아있으나 14일 이후부터 감소하기 시작하여 21일 이후에는 완전히 분해되어 배출되는 것을 확인 하였다[20]. GUARDIX-SG^Ⓡ^ 는 이미 백서에서 유착방지에 대한 효과를 확인하였고 임상에서는 척추와 갑상선 절제술 환자에게 적용하여 안전성과 유착방지 효과가 있는 것을 확인하였다[18-20, 22].

이와 같은 근거로, 본 시험은 변형 액와부림프절곽청술을 시행한 환자 중 변형 근치 유방절제술 및 유방보존수술을 시행한 환자를 대상으로 수술 후 12개월간 추적관찰을 시행할 예정이며, GUARDIX-SG^Ⓡ^의 상지기능 장애 예방 효과와 유방암 수술에서 GUARDIX-SG^Ⓡ^ 의 안전성을 평가하고자 한다.

**7. 임상시험용 의료기기의 사용목적(대상질환 또는 적응증)**

**7-1. 임상시험용 의료기기의 사용목적**

유방암으로 변형근치유방절제술 혹은 유방보존수술 및 액와부림프절곽청술을 시행 받는 환자를 대상으로 수술부위에 GUARDIX-SG^Ⓡ^를 도포한 시험군과 도포하지 않은 대조군 간의 어깨 관절 가동범위의 차이를 확인하여 GUARDIX-SG^Ⓡ^가 유방암 수술 후 어깨 관절 운동 제한을 예방하는 것에 대한 유효성을 알아보고자 한다.

**7-2. 대상질환**

유방암

( 액와부림프절곽청술 피험자 중 변형근치유방절제술 혹은 유방 보존 수술을 시행한 자 )

**8. 피험자 선정기준, 제외기준, 목표한 피험자 수 및 그 근거**

**8-1 피험자 선정 기준**

1) 20세 이상의 성인

2) 유방암으로 진단을 받은 환자

3) 유방암 환자 중 변형근치유방절제술 혹은 유방보존수술을 받으면서 액와부림프절곽청술을 시행한 환자

(수술 전 항암치료를 받은 환자도 수술 시 액와부림프절곽청술을 시행하는 경우는 대상환자 에 포함)

**8-2 피험자 제외 기준**

1) 상피내암

2) 4기 (Stage IV)

3) 감시림프절 생검술만 시행하는 환자

4) 유방전절제술 및 동시복원 시행환자

5) 수술 전 기타 어깨 질환으로 어깨 관절 운동의 장애가 있는 경우

6) 이전의 동측 유방암 혹은 액와부의 수술 기왕력이 있는 경우

7) 임신 중이거나 수유중인 환자

**8-3 목표한 피험자 수 및 산출 근거 [28]**

본 연구는 Poloxamer와 alginate로 제조된 유착방지제 GUARDIX-SG를 유방암으로 변형 근치 유방절제술 혹은 유방보존수술 및 액와부림프절곽청술을 시행 받을 환자에서 어깨 관절 가동범위에 미치는 영향을 알아보는 시험이다.

문헌에서는 어깨관절가동범위가(ROM) 10도 초과 차이 나는 것을 임상적으로 유의하다고 보고 있으며[17], 실험군과 대조군을 비교하여 10도 초과 차이 나는 경우 유의한 차이가 있다고 판정한다. 이에 정밀도 0.025, power 90%, 표준편차 10[25]하에 탈락률 10% 고려 시 각 환자군당 96명으로 정하여 총 192명의 피험자 수를 산출하였다. Pass Software를 사용하여 피험자 수를 산출하였다.

* 본 연구 가설은 다음과 같다.

**H_0_ : D ≤ 10 H_1_ : D 〉 10 (D = 실험군 ROM Mean – 대조군 ROM Mean)**

* 산출근거 : 유효한 피험자 수를 산정하기 위하여 다음을 가정한다.

① 유의수준 α = 0.025

② 시험군의 예수는 대조군 예수의 1배이다.

③ 제 2종 과오(β)는 0.1으로 하여 검정력 90%를 유지한다.

④ 두 군의 평균값의 차이 : 10도


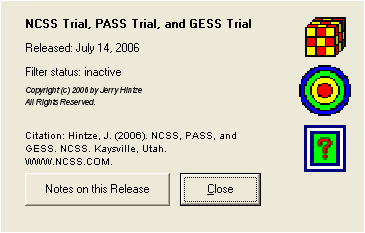


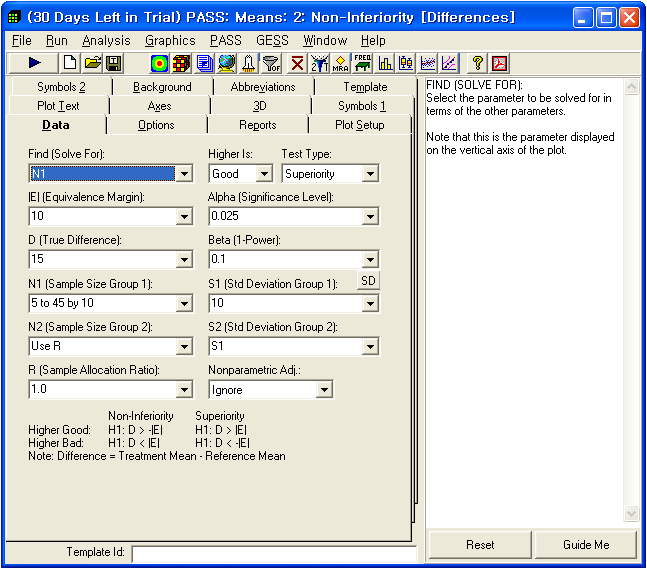


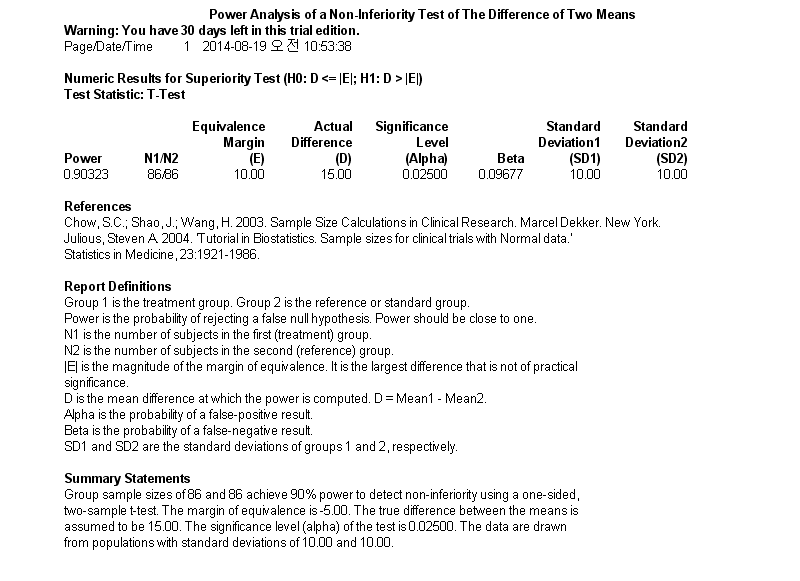


| Power | N1/N2 | Equivalence Margin  (E) | Actual  Difference  (D) | Significance  Level  (Alpha) | Beta | Standard  Deviation1  (SD1) | Standard  Deviation2  (SD2) |
| --- | --- | --- | --- | --- | --- | --- | --- |
| 0.90022 | 23/23 | 5 | 10 | 0.025 | 0.09978 | 5 | 5 |
| 0.80044 | 17/17 | 5 | 10 | 0.025 | 0.19956 | 5 | 5 |
| 0.90004 | 86/86 | 5 | 10 | 0.025 | 0.09996 | 10 | 10 |
| 0.80003 | 64/64 | 5 | 10 | 0.025 | 0.19997 | 10 | 10 |
| 0.90022 | 191/191 | 5 | 10 | 0.025 | 0.09978 | 15 | 15 |
| 0.80044 | 143/143 | 5 | 10 | 0.025 | 0.19956 | 15 | 15 |
| 0.91250 | 23/23 | 10 | 15 | 0.025 | 0.08750 | 5 | 5 |
| 0.80704 | 17/17 | 10 | 15 | 0.025 | 0.19296 | 5 | 5 |
| 0.90323 | 86/86 | 10 | 15 | 0.025 | 0.09677 | 10 | 10 |
| 0.80146 | 64/64 | 10 | 15 | 0.025 | 0.19854 | 10 | 10 |
| 0.90022 | 191/191 | 10 | 15 | 0.025 | 0.09978 | 15 | 15 |
| 0.80044 | 143/143 | 10 | 15 | 0.025 | 0.19956 | 15 | 15 |

10%의 피험자가 중도 탈락한다고 예상을 할 때, 필요한 총 피험자의 수는 192명으로 시험군 96명, 대조군은 96명이다.

| 목표한 피험자 수 | 시험군 | 대조군 | 총피험자수 |
| --- | --- | --- | --- |
| 최종 유효성 평가 피험자 수 | 86 | 86 | 172 |
| 탈락율(10%)을 포함한 피험자 수 | 96 | 96 | 192 |

**9. 임상시험기간**

식약처 승인 후 IRB 승인일로부터 30개월

* 피험자 모집기간 : 12개월

* 임상관찰 및 시험수행기간 : 12개월

* 통계처리기간 : 4개월

* 결과보고서 작성기간 : 2개월

**10. 임상시험방법**

**10.1 임상시험용 의료기기**

(1) 시험 의료기기 명칭

1) 품목허가번호 : 제09-826호 (코드 : BM2102JO)

2) 품목명 : 심부체강창상피복재

3) 제품명 : GUARDIX-SG

4) 일반명 : 유착방지제(용액형)

(2) 원자재 성분

Poloxamer, Sodium alginate

(3) 형상 및 구조

1) 형상

GUARDIX-SG 용액은 멸균된 비발열성, 무색, 투명한 점성의 poloxamer/sodium alginate 혼합용액이며, 수술 후 상처 면에 일시적인 점조성/윤활성 코팅을 제공하며, pre-filled syringe에 주입하여, 포장되어 멸균된 상태로 제공됨.


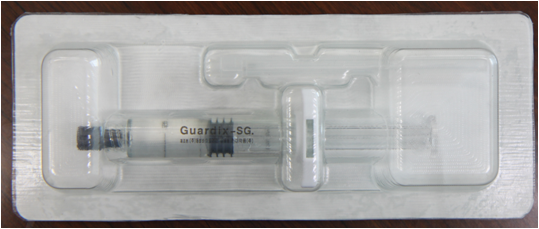

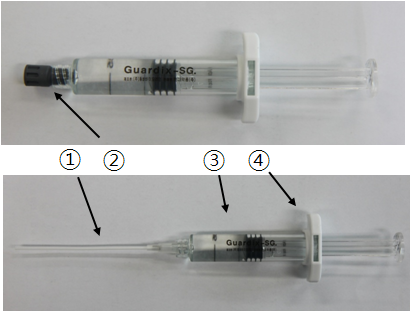
가) 외관사진

나) 외관설명

① 카테터 튜브 : Guardix-SG 용액을 수술 후 상처 면에 근접하여 코팅시킬 수 있도록

해주는 튜브이다.

② 마개 : 주사기의 용액이 흘러내리지 않게 보호하며 밀봉하는 마개이다.

③ 주사기 : Guardix-SG 용액을 담는 용기이다.

④ 핑거그립(손가락걸이)

2) 치수 및 중량

가) 외관치수

5 ㎖, 10 ㎖ 용량의 pre-filled syringe(cycloolefine copolymer)에 3 ㎖, 6 ㎖의 GUARDIX-SG 용액이 담겨져 있으며, syringe의 tip은 마개로 닫힌 채 폴리에스터 고정용 포장 용기에 넣어지며, 용액 주입 시 가이드용으로 폴리에틸렌테레프탈레이트(테프론)의 카데터가 함께 넣어지고, Tyvek과 함께 접합하여 포장된다.

나) 중량 : 3 ㎖, 6 ㎖

**10.2 대조시험용 의료기기**

비활성 대조군으로 대조시험용 의료기기는 사용하지 않는다.

[현재 유방암으로 변형근치유방절제술 혹은 유방보존수술 및 액와부림프절곽청술을 시행 받는 환자의 경우 수술 부위에 유착방지제를 사용하는 것이 일반적이지 않아 대부분이 유착방지제 도포 없이 수술되어지고 있어 대조군으로 어떠한 제품을 사용하지 않는 것이 안전하며, 대조군으로 사용할 수 있는 의료기기가 현재는 없는 상태로 비활성 대조군으로 임상시험을 설정하였다.]

**10.3 임상시험방법**

(1) 임상시험 설계

서울아산병원에서 유방암으로 변형근치유방절제술 혹은 유방보존수술 및 액와부림프절곽청술을 시행 받는 환자 중 자의에 의해 피험자 동의서에 서명한 피험자는 스크리닝 검사를 실시하여 피험자 적합성 평가를 실시한다. 이때 선정 제외 기준에 적합한 피험자는 수술일에 병원에 방문하여 변형근치유방절제술 혹은 유방보존수술 및 액와부림프절곽청술을 시행한 후 무작위배정표에 따라 시험군 또는 대조군 중 한 군으로 배정 된다.

ν 시험군 : Guardix-SG

ν 대조군 : 사용하지 않음

배정된 피험자는 군에 따라 수술부위에 Guardix-SG를 적용하는 경우와 사용하지 않는 경우로 나눠지며 임상시험용 의료기기는 수술 시에만 적용된다. 수술 후 7일, 3개월, 6개월, 12개월에 각각 해당되는 검사를 진행한다.

(2) 피험자 무작위 배정

본 임상시험은 시험군과 대조군 처치의 할당에서 개입될 수 있는 Bias를 줄이고, 두 군의 비교 성을 높이기 위하여 블록 무작위 배정법을 실시한다. 유방절제수술 환자와 부분절제 수술을 받은 환자에서 올 수 있는 ROM운동범위의 정확한 차이를 비교하기 위해 두 수술 간에 같은 환자의 비율로 무작위 배정을 한다.

무작위 배정법은 액셀의 랜덤함수(RAND)를 이용하여 각 피험자에 소수점 4자리의 난수를 발생시켜 블록 무작위 배정을 하며 각 배정표는 봉인되어 시험자에게 전달 되도록 한다. 임상시험 의뢰자는 무작위 할당된 목록에 따라 순서대로 대조군과 처치군이 기입된 눈가림 표를 공급한다. 이후 본 임상시험에 적합한 피험자에게 피험자 번호를 배정하는 것은 해당 임상시험기관에서 이루어지며 임상시험기관에서는 동의서 수령 및 선정을 위한 검사를 시행하는 피험자에게 스크리닝 번호를 부여한다. 스크리닝을 위한 모든 과정을 마치고 해당 피험자가 본 임상시험의 대상자로 적합한 경우에 무작위 배정번호를 순차적으로 부여하게 되며 임상시험에서 탈락한 피험자의 스크리닝 번호와 무작위 배정번호는 계속 유지되고, 탈락하는 경우 그 번호도 같이 탈락처리 된다. 새로운 피험자는 반드시 새로운 피험자 스크리닝 번호와 무작위 배정번호를 할당받아야 한다.

(3) 맹검해제

어떠한 경우에도 시험도중에 맹검이 해제되어서는 안 된다. 다만, 피험자가 위급상황에 빠져서 피험자가 시험군인지 대조군인지를 알아야 위급상황에 대처할 수 있는 경우에는 적절한 과정을 거쳐 맹검을 해제하며, 이러한 일은 문서로 남겨야 한다.

(4) 수술 방법 및 임상시험용 의료기기 적용방법

시험군과 대조군 모두 유방암의 치료에 대한 표준술식으로 액와부림프절곽청술 시행 환자 중 변형 근치 유방 절제술 또는 유방보존수술 시행 환자에 한해서 수술 시행 후 총 주입용량 3ml 적용한다. 이때 유착방지제의 도포는 시험자에 의해 직접 이루어지게 되며 대조군의 경우는 유착방지제를 적용하지 않으며 두군 모두 수술 후 치료와 처치는 동일하다.

**11. 관찰항목 ∙ 임상검사항목 및 관찰검사 방법**

**11-1 관찰 항목 및 방법**

(1) 피험자 동의 및 인구학적 조사

임상시험에 들어가기 전, 본 임상시험의 목적과 내용에 대하여 피험자에게 상세히 설명하고, 서면 동의를 받고, 증례기록서에 인구학적 정보(나이, 성별, 체질량 지수)를 조사하여 서면 동의 부 및 동의 일자와 피험자 이니셜, 체중, BMI와 대한 자료를 기록한다. 서명된 동의서의 사본은 피험자에게 제공하며 지속적으로 임상시험에 대한 사항을 확인 할 수 있도록 한다.

(2) 과거력 및 Physical function

수술 전 병기 및 병력, 어깨 관절부위 기저질환 관련 정보, 일반혈액검사, 어깨 관절 가동범위, 상지운동 수행 능력 평가, 동작 시 통증평가에 대한 자료를 조사하여 기록한다.

(3) 피험자 적합성 평가(선정/제외 기준 확인)

피험자 동의여부와 인구학적 조사, 병력, 수술 전 조직학적 진단 등을 바탕으로 선정/제외기준을 확인하여 적합한 피험자가 선정되었는지 평가하여 기록한다.

(4) 수술과 관련된 관찰 항목

-수술관련 기초 정보

수술일, 수술명, 수술 후 병리조직학적 진단, 최종 병기(TNM stage), 수술 후 보조치료(항암화학요법, 항호르몬치료, 표적치료, 방사선치료)

-안전성 평가

수술관련 및 입원 중 합병증(이상반응), 수술 후 배액량 측정, 감염의 징후 관찰, 전혈검사(POD #7)

(5) Physical function (수술 전, 수술 후 3, 6, 12개월)

-유효성 평가

어깨 관절 가동범위(Abduction, Horizontal abduction, Flexion, External rotation), 상지운동 수행 능력 평가, 동작 시 통증평가, 체성분분석을 이용한 림프부종의 평가

**11-2 유효성 평가항목 및 방법** [6], [8], [9], [12], [13], [17], [23], [24], [25], [26], [27]

(1) 어깨 관절 가동범위

Shoulder ROM은 4가지의 active range of motion을 측정하며, 측정에 universal full-circle manual goniometer 가 쓰임.


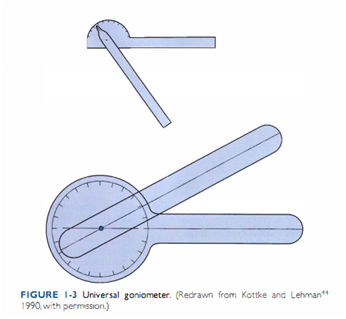


**<Shoulder flexion >**

Abduction, flexion 은 neutral anatomical position에서 coronal, scapular plane and sagittal planes으로 측정합니다.


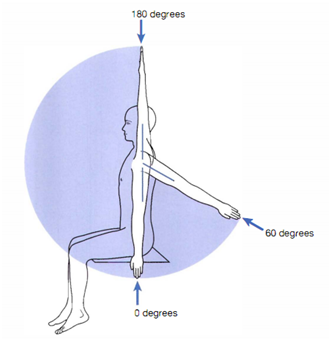


**<Abduction>**


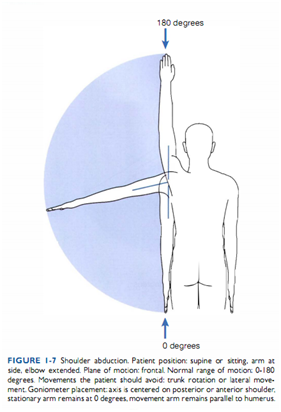


**<external rotation>**

External rotation은 눕거나 선 자세에서 shoulder 90도 abduction, elbow 90도 flexion, forearm pronation 한 자세에서 측정합니다. 선 자세에서 측정 시 forearm이 바닥에 horizontal하고 palm이 바닥을 향한 상태에서 측정합니다.


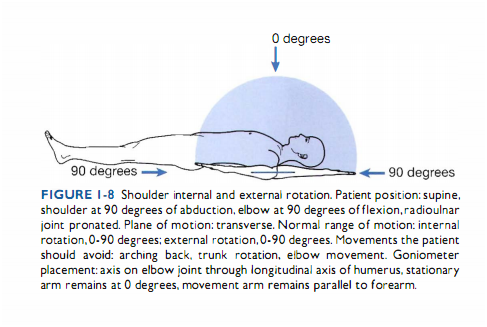


<**Horizontal abduction**>

shoulder 90도 abduction, elbow 90도 flexion에서 horizontal plane에서 최대한 abduction한 각도를 측정

(2) 상지운동 수행능력 평가(설문도구 – DASH)

**별도로 첨부함

(3) 동작시 통증평가

피험자의 통증 정도를 검증된 측정도구인 VAS(visual analogue scale)를 이용하여 0(통증없음)에서 100(최고 통증) 중에서 환자가 직접 표시하도록 하고, 시험담당자는 이를 자로 재어서 증례기록서에 기재한다.

**별도로 첨부함

(4) 체성분분석을 이용한 림프부종의 평가

체성분분석기인 인바디S10을 이용하여 체성분분석을 하여 수술한 측 팔의 림프부종 정도를 평가한다.

**11-3 안전성 평가항목 및 방법**

(1) 실험실적 검사

혈액학적 검사 – WBC, Neutrophil

(2) 이상반응 평가

시험자 또는 시험담당자는 이상반응에 대하여 피험자가 자발적인 보고를 하도록 수시로 교육해야 하며, 방문 시 면담 및 문진 등에 진료를 통하여 이상반응을 확인한다.

(3) 동작 시 통증평가

(4) 수술 후 배액량

배액량 (ml)단위로 조사하여 기재하도록 한다.

(5) 예상되는 부작용

임상시험에 사용되는 의료기기 사용 후 나타날 수 있는 예상되는 부작용으로 감염의 경우 수술 후 7일째 백혈구 혈액검사(WBC) 및 진찰을 통해 확인하고 알레르기 반응 (전신성 발진, 호흡곤란, 부종, 통증, 염증) 또한 진찰을 통해 관찰 후 의료기기와의 인과관계를 확인한다.

**11-4 관찰검사 시기 및 진행일정**

피험자는 스크리닝 검사를 통해 선정/제외 기준에 적합함이 확인되면 시험군 및 대조군으로 무작위 배정되어 임상시험을 진행하게 된다.

각 방문 별로 시행되는 관찰 항목은 다음과 같으며 연구일정표에 따라 진행하도록 한다.

**연구일정표**

| 구분 | 처치 및 관찰기간 | | | | | |
| --- | --- | --- | --- | --- | --- | --- |
| 방문수 | Visit 1 | Visit 2^1^ | | Visit 3 | Visit 4 | Visit 5 |
|  | 수술전 | 수술  직후 | 수술후  7일 | 수술후  3개월 | 수술후  6개월 | 수술후  12개월 |
| Visit Window (일) |  |  | ± 2 | ± 14 | ± 14 | ± 30 |
| 선정/제외 기준 | ○ |  |  |  |  |  |
| 서면 동의서 | ○ |  |  |  |  |  |
| 기초정보 및 의학적 병력 | ○ |  |  |  |  |  |
| 혈액 검사^2^ | ○ |  | ○ |  |  |  |
| 배액량 측정³ |  | ○ | ○ |  |  |  |
| 체성분분석 | ○ |  |  | ○ | ○ | ○ |
| 시험의료기기 처방 |  | ○ |  |  |  |  |
| 어깨관절가동범위 측정 | ○ |  |  | ○ | ○ | ○ |
| 상지운동 수행능력 평가 | ○ |  |  | ○ | ○ | ○ |
| 통증평가 | ○ |  |  | ○ | ○ | ○ |
| 이상반응평가 |  |  | ○ | ○ | ○ | ○ |

1. visit 2 해당되는 혈액검사 및 이상반응 평가는 퇴원일에 이상반응 평가를 진행하거나 수술 후 7일에 재방문하여 평가할 수 있다.

2. 혈액 검사 - WBC, Neutrophil

수술 후 7일에 시행 예정인 혈액검사는 수술 후 발열이나 수술부위의 발적 등 이상반응이 있는 경우에만 백혈구 및 염증 수치 증가여부를 확인하기 위해 CBC, CRP 등의 검사를 시행하며 수술 후 7일 이내의 검사결과를 사용할 수 있다.

3. 배액량측정 - 수술 후 7일까지 2일 간격으로 하고 퇴원 시 마지막 측정을 하고 이후에는 종료한다.

① 방문 1 (스크리닝 방문)

ν 피험자 동의서 취득

ν 피험자 기초정보, 의학적 병력 및 어깨 관절부위 기저질환 관련 조사

ν 수술 전 조직학적 진단

ν 실험실적 검사 실시

ν Physical function (어깨 관절 가동범위 : Abduction, Horizontal abduction, Flexion, External rotation), 상지운동 수행 능력 평가, 동작 시 통증평가)

ν 체성분분석

ν 시험참여에 대한 피험자 적합성 평가

② 방문 2(OPD~POD #7)

ν 시험의료기기 처방(시험군, 대조군 무작위 배정)

ν 병리조직학적 진단 및 최종병기

ν 수술후 보조치료(항암화학요법, 항호르몬치료, 표적치료, 방사선치료)

ν 수술 후 배액량

ν 실험실적 검사 실시(POD #7)

ν 이상반응 평가

③ 방문 3, 4, 5(수술 후 3개월, 6개월, 12개월)

ν Physical function (어깨 관절 가동범위 : Abduction, Horizontal abduction, Flexion, External rotation), 상지운동 수행 능력 평가, 동작 시 통증평가

ν 체성분분석

ν 이상반응 평가

**12. 예측 부작용 및 사용상의 주의사항**

**12-1 일반적인 주의사항**

1) 본 제품은 멸균제품이므로 시술하는 동안 멸균상태가 유지되도록 하고 유효 날짜가

경과하면 사용하지 말 것.

2) 사용 전에 내용물을 점검한 후 포장 및 용기의 손상이나 습기의 흔적이 보이면 사용하

지 말 것.

3) GUARDIX-SG 용액을 수술 부위에 투입하기 이전에 수술 의사는 여분의 액체는 흡인기

로 빨아들여 제거할 것.

**12-2 취급 시 주의사항**

1) GUARDIX-SG 용액은 실온에서 보관하여 사용되어야 한다.

2) GUARDIX-SG 용액을 체온까지 데워 젤이 될 경우에도 사용할 수 있다.

3) GUARDIX-SG는 멸균되어 제공되며, 재 멸균하여 사용할 수 없다.

4) 일회용이므로 사용 후 남은 용액을 폐기해야 하며, 재사용 하면 안 된다.

**12-3 금기사항**

1) 투여금지 환자

ㄱ. GUARDIX-SG 용액은 수술부위에 감염이나 오염이 된 환자에게 적용되지 않도록 한다.

ㄴ. GUARDIX-SG 용액은 제품 성분에 대하여 과민증이 있는 환자들에게 이용하면 안 된다.

2) 신중한 투여

ㄱ. GUARDIX-SG 용액은 다른 유착방지 제품, 흡수성 지혈제, 다른 약물들과 조합하여

사용하는 경우, 그 안전성 및 유효성은 동물시험에서 확인되지 않았다.

ㄴ. GUARDIX-SG 용액은 임신기간 중에는 사용이 추천되지는 않는다. GUARDIX-SG 용액

사용 후 첫 월경 주기가 끝날 때까지는 임신을 피하기를 권고한다.

**12-4 유착방지제 사용 시 예측 부작용**

감염 : 발열, 백혈구수 증가, 알레르기 반응 : 전신성 발진, 호흡곤란, 부종, 통증, 염증

* Guardix-SG 유착방지제는 손상된 조직으로 섬유화 세포들이 침투하지 못하도록 물리적인 barrier 역할을 통해 유착을 방지하는 제품으로 의료기기로 인해 예측되는 부작용의 정도는 같은 증상이더라도 환자 및 상태에 따라 경증, 중증도, 중증으로 차이날 수 있어 그 정도를 기록한다.

(1) 경증(mild) : 부작용의 정도가 경미한 경우

(2) 중증도(moderate) : 부작용의 정도가 중증도인 경우

(3) 중증(Severe) : 부작용의 정도가 심한 경우

**12-5 수술 시 및 수술 후 예측 부작용**

출혈, 창상감염, 피부 피판 괴사, 어깨 관절 범위 감소, 환상 유방 증후군, 장액종, 신경 손상 및 감각 이상, 림프부종, 목, 등, 팔의 통증과 불편감, 수술 부위 피부가 조이는 느낌 등

**12-6 마취 시 예측 부작용**

간독성, 신독성, 핍뇨, 다뇨, 마이오글로빈뇨, 저혈압, 고혈압, 서맥, 빈맥, 혈압변동, 부정맥, 심전도 이상, 심박출량 저하, 심실성기외수축, 상실성 기외수축, 심방성 기외수축, 방실블록, 이단맥, 기침, 호흡기 장애, 무호흡, 천식, 저산소증, 후두경련, 고체온증, 저체온증, 악성고열증, 초조, 어지러움, 경련양운동, 흥분, 착란, 근강직, 구토, 오심, 떨림, 두통, 피로감, 아낙필락시스양 또는 아나필락토이드 반응과 같은 알러지 반응, 백혈구 증가, 백혈구 감소 및 일시적 혈당 증가, 근육통, 탈력감, CPK상승 등

**13. 중지 및 중도탈락 시 처리 방법**

시험을 중지하거나 시험에서 탈락되는 경우, 중지 · 중도 탈락한 예로부터 중지 · 중도 탈락 시까지 얻은 시험의 결과는 최종 평가 시 평가 가능한 항목에 대해서 검토될 수 있다.

**13-1 중지 기준**

다음 사례가 발생한 경우 임상시험실시기관에서 해당 임상시험계획에 기초하여 임상시험의 계속 유무를 검토한다.

1) 시험자는 임상시험과정에서 관찰된 결과에 비추어 임상시험을 지속하는 것이 현명하지 않다고 판단될 경우 시험의 일부 혹은 전부를 중지시킬 수 있다.

2) 안전성 혹은 관리상의 이유로 임상시험의 일부 혹은 전부를 중지시킬 수 있다.

3) 임상시험과정에서 관찰된 결과에 따라 임상시험을 지속하는 것이 현명하지 않다고 판단될 경우 시험자는 임상시험위원회에 임상시험 중지요청을 하여야 하고, 임상시험심사위원회의 결정에 따라 임상시험을 중지할 수 있다.

4) 임상시험계획의 변경이 필요하지만 시험기관이 이에 대응할 수 없을 때 임상시험을 중지할 수 있다.

5) 시험기관의 임상시험심사위원회의 의견에 기초하여 시험기관 장이 수정지시를 내렸을 때 이를 승낙할 수 없는 경우 임상시험을 중지할 수 있다.

6) 임사시험을 계속 할 수 없다는 시험기관의 임상시험심사위원회의 의견에 따라 시험기관장이 임상시험의 중지를 지시한 경우 임상시험을 중지할 수 있다.

7) 시험기관이 KGCP, 본 임상시험계획서 또는 임상시험계약서와는 다르게 중대하거나 계속적인 위반행위를 한 경우 임상시험을 중지할 수 있다.

**13-2. 중지 시의 처리방법**

1) 임상시험의 중지를 결정한 경우 신속하게 그 취지 및 이유를 시험기관의 장에게 문서로 통지한다.

2) 시험기관의 장은 임상시험의뢰자로부터 임상시험을 중지한다는 취지의 통지를 접수한 경우 신속하게 그 취지와 이유를 임상시험책임자 및 임상시험심사위원회에 문서로 통지한다.

3) 시험책임자는 의뢰자로부터 시험기관의 장을 통하여 임상시험의 중지와 관련된 통지를 접수한 경우 피험자에게 신속하게 그 취지를 통보하고 적절한 치료 및 사후처리를 보증한다.

4) 임상시험을 중지한 경우에 피험자에 대하나 대응에 대해서는 본 시험계획서 내의 중지시에 처치에 따른다.

5) 시험책임자가 의뢰자와의 사전합의 없이 임상시험을 조기종료 또는 일시중지 하였을 경우, 시험책임자는 이 사실을 심사위원회에게 즉시 알리고, 조기종료 또는 일시중지에 대한 상세한 사유서를 제출하여야 하다.

6) 심사위원회가 임상시험을 조기종료 또는 일시중지 시켰을 경우, 시험책임자는 이 사실을 의뢰자에게 즉시 알리고, 조기종료 및 일시중지에 대한 상세한 사유서를 제출하여야 한다.

**13-3. 중도 탈락 (Withdrawal)**

등록이란 본 임상시험에 참여한(피험자 동의서에 서명한) 피험자가 무작위배정(피험자 번호 부여)을 받은 것을 말한다. 중도탈락이란 시험의료기기를 한 번 이상 적용하였으나, 어떠한 이유로든 시험과정을 종료하지 못한 자.

**13-4. 중도 탈락 기준**

1) 피험자의 상태 또는 기타 이유에 따라 필요한 검사 또는 실시가 불가능하다고 판명된 경우

2) 피험자가 임상시험 진행이 어려울 정도의 이상반응(AE), 이상의료기기반응(ADE), 중대한 이상반응(SAE)이 나타나 시험의 지속이 곤란하다고 판단되는 경우

3) 중대한 임상시험계획서의 위반이 발생한 경우

- 유효성 평가에 영향을 미칠 수 있는 선정기준의 위반

4) 피험자 또는 법적대리인이 동의 철회를 통해 임상시험의 중단을 요구하는 경우

5) 피험자의 추적이 안 되는 경우

6) 기타 시험자가 시험을 중지하여야 한다고 판단할 경우(임신 등)

**13-5. 중도 탈락 시의 처리방법**

1) 탈락 예에 대해서는 필요에 따라 적절한 처치 또는 치료를 행하며 필요한 경과를 관찰 하고 이를 기록한다. 중도 탈락 사유를 증례기록서에 기록해야 한다.

2) 탈락 예는 탈락시점까지 모든 검사결과와 함께 탈락일, 탈락에 대한 이유, 탈락 시의 처치와 경과를 증례기록서에 기입한다.

3) 피험자가 임상시험 도중에 내원하지 않는 경우에는 편지, 면회, 전화 등을 통하여 가능한 그 이유 및 향후 경과를 추적 조사하여 증례기록서에 경과를 기입한다.

4) 탈락 예에 대해서는 새로운 피험자로 대체되지 않는다.

5) 피험자가 임상시험 완료 이전에 임상시험의 참여를 그만 둘 경우 그 이유를 제시할 의무는 없지만 시험자는 피험자의 권리를 충분히 고려하면서 해당 사유를 확인하기 위한 노력을 하여야 한다.

6) 탈락시점에서 실시된 유효성 및 안전성 평가(이상반응 유무, 병용약물) 결과가 있다면, 분석에 포함시키는 것이 결과의 신뢰성에 비뚤림에 영향을 미치지 않는다면 모두 포함시켜 분석하는 것으로 한다.

7) 임신 환자의 경우 탈락이 된 이후에도 추적관찰하고 윤리적인 목적으로만 자료를 수집한다.

**13-6 임상시험계획서 위반에 대한 처리**

임상시험을 진행하는 동안 임상시험계획서를 위반하였음이 알려진 경우 해당 시험자가 임상시험을 지속해야 할지, 중단해야 할지를 결정해야 한다. 증례기록서(case report form)에 임상시험계획서 위반내용 및 그 사유를 자세하게 기록해야한다. 이때 유효성 및 안전성에 미칠 영향에 대한 연구자의 판단에 따라 탈락 또는 계속 진행을 결정하도록 한다.

**14. 성능의 평가 기준**

**14-1. 유효성의 평가변수** [6], [8], [9], [12], [13], [17], [23], [24], [25], [26], [27]

수술 전과 수술 후의 Physical function 측정한다.

- 어깨 관절 가동범위 (Abduction, Horizontal abduction, Flexion, External rotation)

- 상지운동 수행 능력 평가

- 동작 시 통증평가

**14-2. 안전성의 평가변수**

실험실 검사의 이상소견 및 이상반응 발생여부

**14-3. 기타 평가변수**

수술 후 혈액의 배액량, 수술 후 보조치료(항암화학요법, 항호르몬치료, 표적치료, 방사선치료)

**15. 유효성의 평가기준, 평가방법, 해석방법(통계분석방법)**

**15-1 유효성의 평가방법 및 해석**

1) 연구자 및 피험자 설문지로 평가

2) 1차 유효성 평가변수 분석

어깨관절가동범위를 ROM(range of motion) 4가지 상태(Abduction, Horizontal abduction, Flexion, External rotation)에서 universal full-circle mamual goniometer을 이용하여 수술 전, 수술 후 3개월, 6개월, 12개월에 각각 값(각도)을 측정한다. 측정 된 값은 각 시점에서의 군간 차이와 수술 전 ~ 수술 후 3개월, 수술 전 ~ 수술 후 6개월, 수술 전 ~ 수술 후 12개월 사이의 군간 변화 차이를 One side, Two sample student t-test를 실시하여 ROM 각각의 상태별 유의성을 비교 분석한다. 두 군의 차이가 유의하게 10도 초과일 경우 (즉, 95% 신뢰구간의 하한값이 10 이상인 경우) 시험군이 우월하다고 판단한다.

(임상적으로 두 군 간의 값은 비교하여 10도 초과 차이나는 경우 유의한 차이가 있다고 판정한다.)

3) 2차 유효성 평가변수 분석

- 상지운동 수행능력 평가(설문도구 – DASH(disability of the arm, shoulder and hand)

DASH 설문지는 총 30문항으로 구성되어 있으며 피험자가 설문지에 답변한 것을 점수로 계산한다.

**DASH장애/증상점수=[(답변된 점수의 합)/n-1]X25**

* n은 답변된 문항의 수를 의미하며, 세 문항 이상에 답변이 없다면 DASH장애/증상점수를 계산 할 수 없다.

측정 된 점수는 각 시점에서의 군간 차이와 수술 전 ~ 수술 후 3개월, 수술 전 ~ 수술 후 6개월, 수술 전 ~ 수술 후 12개월 사이의 군간 변화 차이를 One side, Two sample student t-test를 실시하여 유의성을 비교 분석한다.

- 동작시 통증평가

피험자의 통증 정도를 검증된 측정도구인 VAS(visual analogue scale)를 이용하여 100mm 선상에 0(통증없음)에서 100(최고 통증) 중에서 환자가 직접 표시하도록 하고, 시험담당자는 이를 자로 잰다. 측정 된 값은 각 시점에서의 군간 차이와 수술 전 ~ 수술 후 3개월, 수술 전 ~ 수술 후 6개월, 수술 전 ~ 수술 후 12개월 사이의 군간 변화 차이를 One side, Two sample student t-test를 실시하여 유의성을 비교 분석한다.

**15-2 안전성 평가분석**

1) 이상반응

시험기간 중 발생한 모든 이상반응에 대하여 시행한다. 이상반응의 발현율, 중도탈락의 원인이 된 이상반응의 발현율, 중대한 이상반응의 발현율을 처리군별로 요약하며, 모든 임상기간동안 발현한 이상반응에 대해 Chi-square test 또는 Fisher’s exact test를 이용하여 검정한다. 이상반응의 발현율은 모든 이상반응에 대한 발현율 및 임상시험용 의료기기와 관련이 있는 이상반응의 발현율을 제시한다.

2) 실험실적 검사

연속형 자료일 경우 기저점에서의 기술통계량(평균, 표준편차, 중앙값, 최소값, 최대값)을 제시하고 자료의 정규분포 여부를 검정하여 One side, Two sample student t-test를 이용하여 두 군간 비교 검정한다. 범주형 자료의 경우 기술통계량(빈도, 백분율)을 제시하고, Chi-square test 또는 Fisher’s exact test를 이용하여 두 군간 비교 검정한다. 수술 전과 수술 후 7일의 검사 수치가 군간에 차이가 있는지 확인하고 차이가 있으면 정상범위 안에서의 차이인지 아닌지를 확인한다.

① 평가 항목 : 이상반응(Visit2, Visit3, Visit4, Visit5) 및 혈액 검사(Visit1, Visit2)

② 평가 기준 : 시험군의 결과값과 대조군의 결과값을 비교 분석하였을 때 유의하지 않으면 시험용 의료기기에 의한 안전성은 확인 된 것으로 한다.

**15-3 통계분석**

1) 방법 : One side, Two sample student t-test, Chi-square test, Fisher’s exact test

2) 주 분석 : ITT군

3) 부가적인 분석 : PP군

4) 검정력 : 80%

* 임상시험에서 중지․탈락된 피험자는 유효성 평가 통계처리 시 순응군(per protocol :

PP군)에서 제외하며, 배정된 대로의 분석군(intention to treat : ITT군)에서는 결측치

처리 방법에 따른다.

5) 통계분석의 일반적 원칙

본 임상시험의 피험자로부터 얻어진 자료는 크게 ITT(Intention To Treat)군과 PP(Per Protocol)군의 두 가지 형태로 분석한다.

ITT군은 무작위 배정되어 최소한 한 번이라도 시험제품을 복용한 피험자로부터 얻어진 자료를 모두 분석에 포함한다. 단, 1차와 2차 유효성 평가 변수 결측치 발생 시 유효성 평가변수 분석에서 해당 피험자는 제외하며, 안전성 평가변수만 ITT군에 포함한다.

PP군은 ITT군에 포함되는 피험자 중 임상시험 계획서대로 완료한 피험자로부터 얻어진 자료를 분석에 포함시킨다. 그리고 유효성 평가에 영향을 줄 수 있는 중대한 임상시험계획서 위반(제외기준 2. 유방암으로 진단받은 피험자, 각 Visit(방문)1, 2을 누락한 자, 무작위 배정이 잘못된 피험자, 유효성 평가에 영향을 줄 수 있는 중대한 이상반응이 발생한 피험자는 PP군에서 분석을 제외하고 ITT군으로 분석을 시행한다.

유효성 평가는 원칙적으로 ITT군과 PP군을 모두 실시하며, 분석결과가 다른 경우에는 ITT군을 주 분석법으로 하고, PP군 분석법은 보조 분석법으로 분석하여 그 결과를 ITT군과 비교한다. 인구학적 평가와 안전성 평가는 원칙적으로 ITT군을 실시한다.

6) 인구학적 자료

인구통계학적, 의학적 배경인자에 관하여 기술통계량(빈도, 평균, 표준편차, 중앙값, 최소값, 최대값, 백분율)을 계산하고, 두 군 간에 대하여 연속형 자료는 Independent t-test, Wilcoxon’s rank sum test 또는 범주형 자료에 대해서는 Chi-square test, Fisher’s Exact test 등을 이용하여 비교 검정한다.

**16. 이상반응을 포함한 안전성의 평가기준, 평가방법 및 보고방법**

**16-1 안전성의 평가방법**

피험자에 의해 보고되거나 이학검진에 의해 판명된 이상반응에 대하여 중증도와 유형 및 시험군과 대조군의 이상반응 발생률을 평가하기 위한 분석을 수행한다.

1) 이상반응

시험기간 중 발생한 모든 이상반응에 대하여 시행한다. 이상반응의 발현율, 중도탈락의 원인이 된 이상반응의 발현율, 중대한 이상반응의 발현율을 처리군 별로 요약하며, 의약품 부작용 용어 국제 표준 분류체계인 WHO-ART를 사용하여 이상반응을 코딩한 후, Chi-square test 또는 Fisher’s exact test를 이용하여 검정한다. 이상반응의 발현율은 모든 이상반응에 대한 발현율 및 임상시험용 의료기기와 관련이 있는 이상반응의 발현율을 제시한다.

2) 실험실적 검사

연속형 자료일 경우 기저점에서의 기술통계량(평균, 표준편차, 중앙값, 최소값, 최대값)을 제시하고 자료의 정규분포 여부를 검정하여 Independent t-test 또는 Wilcoxon’s rank sum test를 이용하여 두 군간 비교 검정한다. 범주형 자료의 경우 기술통계량(빈도, 백분율)을 제시하고, Chi-square test 또는 Fisher’s exact test를 이용하여 두 군간 비교 검정한다.

**16-2 이상반응 관련 정의들**

1) 이상반응 (Adverse Event, AE)

이상반응(adverse event, AE)이란, 임상시험 중 피험자에게 발생한 모든 의도하지 않은 증후(sign,실험실 실험 결과의 이상 등을 포함한다), 증상(symptom)또는 질병을 말하며, 해당 임상시험용 의료기기와 반드시 인과관계를 가져야 하는 것은 아니다. 시험대상 질환을 비롯한 기존 임상상태의 일간 변동폭이 예견된 정도이고, 임상적으로 유의하지 않은 정도의 악화된 경우에는 보고대상으로 간주되지 않는다.

본 시험의 시작이후에 발생하는 이상반응은 모두 보고한다. 피험자의 시험 참가 시점은 설명 후 동의를 받은 때를 기준으로 한다.(환자동의는 임상시험 계획에 의거한 특별진단 절차나 처치 이전에 얻어야 한다.) 시험 참가 동의 이후에 발생하는 모든 이상반응은 시험용 의료기기의 투여 여부나 관련성 유무에 상관없이 보고하여야 한다.

2) 이상의료기기반응 (Adverse Device Effect, ADE)

임상시험용 의료기기로 인하여 발생한 모든 유해하고 의도하지 않은 반응으로서 임상시험용 의료기기와의 인과관계를 부정할 수 없는 경우를 말한다.

3) 중대한 이상반응 · 이상의료기기반응(Serious AE · ADE)

임상시험에 사용되는 의료기기로 인하여 발생한 이상반응 또는 이상의료기기반응 중에서 다음의 어느 하나에 해당하는 경우를 말한다.

(1) 사망하거나 생명에 대한 위험이 발생한 경우

(2) 입원할 필요가 있거나 입원 기간을 연장할 필요가 있는 경우

(3) 영구적이거나 중대한 장애 및 기능 저하를 가져온 경우

(4) 태아에게 기형 또는 이상이 발생한 경우

4) 예상치 못한 이상 의료기기 반응(Unexpected Adverse Device Effect)

임상시험자자료집 또는 의료기기의 첨부문서 등 이용 가능한 의료기기 관련 정보에 비추어 이상의료기기반응의 양상이나 위해의 정도에서 차이가 나는 것을 말한다.

**16-3 이상반응의 보고**

임상시험 중 발생하는 모든 이상반응을 기록하는 것은 임상시험 책임자와 담당자의 의무이다. 이상반응은 의학 진단 용어로 기록하여야 하며, 이것이 불가능 할 경우 임상시험 책임자 또는 담당자가 관찰하거나, 피험자가 보고한 증상 및 징후에 대한 용어를 기록하여야 한다. 시험용 의료기기 투여 전에 나타난 모든 증상 및 징후는 증례기록서의 기초정보 기록란에 기록하며, 시험용 의료기기 투여 후 나타난 모든 이상반응은 시험용 의료기기의 관련성과 관계없이,

이상반응은 임상시험책임자 또는 임상시험담당자가 평가하여 증례기록서의 이상반응 기록란에 기록하여야 한다.

이상반응을 알아내는 한 가지 일관된 방법으로서 피험자의 자발적인 보고 외에도, 시험용 의료기기 투여 시마다 피험자에게 다음과 같이 원하는 대답을 유도하지 않는 방식으로 질문을 한다. “지난 방문 또는 투여 이후에 다르게 느껴진 바는 없습니까?”

중대하지 않은 이상반응일 경우 연구자가 근거문서와 증례기록서에 이상반응을 작성한다.

중대한 이상반응일 경우 대개 연구자가 알게 된 시점으로부터 24시간 이내에 책임연구자에게 보고해야한다. 또한 임상시험심사위원회에도 해당 규정에 따라 중대한 이상반응을 보고해야하는데, 실시기관에 따라 즉시(24시간 이내)에 보고하거나 중간보고시점에 정리하여 보고할 수 잇다. 그러나 예상하지 못한 이상의료기기반응일 경우 의뢰자와 심사위원회에 즉시 보고하는 것이 원칙이다.

보고 시 연구자는 이상반응 보고서를 작성 후 의뢰자가 있는 경우 시험자는 의뢰자 및 임상심사위원회에 보고한다. 의뢰자는 별지1호 서식에 따라 식품의약품안전처와 임상심사위원회에 보고 후 필요 시 조치를 한다. 의뢰자가 없는 경우 임상심사위원회와 식품의약품안전처에 바로 보고하도록 한다.

가) 임상시험책임자 및 담당자는 다른 시험자, 심사위원회 및 처장에게 중대하고 예상하지 못한 모든 이상반응(이상의료기기반응, 심각한 이상반응/이상의료기기반응 포함한다)을 다음과 같은 기간 내에 “의료기기 시행규칙” 별지 제 35호 이상의료기기반응 신속보고서를 작성하여 가능한 신속히 보고 해야 한다.

① 사망을 초래하거나 생명을 위협하는 경우에는 의뢰자가 이 사실을 보고받거나 알게 된 날로부터 7일 이내. 이 경우 상세한 정보를 최초 보고일로부터 8일 이내에 추가로 보고하여야 한다.

② 그 밖의 중대하고 예상하지 못한 모든 이상반응이 나타난 경우에는 임상시험책임자 및 담당자가 이 사실을 보고받거나 알게 된 날로부터 15일 이내

나) 임상시험책임자 및 담당자는 가)항의 보고와 관련하여 추가적인 안전성 정보를 주기적으로 해당 이상반응이 종결(해당 이상반응의 소실 또는 추적조사의 불가 등)될 때까지 보고하여야 한다.

다) 임상시험책임자는 처장에게 제1항의 규정에 의한 이상반응을 보고하고자 하는 경우에는 식품의약품안전처 “의료기기 시행규칙” 별지 제36호 서식에 의한 이상반응보고서에 ‘3) 안전성 관련 사항의 보고’ 규정에 의하여 보고받은 내용을 첨부하여 제출하여야 한다.

**16-4 이상반응의 중증도 평가**

이상반응의 중증도는 최대 강도(maximal intensity)에 의거하여 아래의 기준에 의해 분류

한다.

1) 경증 (mild) : 피험자의 정상적인 일상생활(기능)을 저해치 않고, 최소한의 불편을 야기

하여, 피험자가 쉽게 견딜 수 있는 경우

2) 중등도 (moderate) : 피험자의 정상적인 일생생활(기능)을 유의하게 저해하는 불편을

야기하는 경우

3) 중증 (severe) : 피험자의 정상적인 일생생활(기능)을 불가능하게 하는 경우

**16-4-1. 이상반응과 시험용 의료기기와의 인과관계 평가 및 평가결과**

임상시험용 의료기기와 이상반응의 인과관계를 평가하는 것은 신속보고 여부를 결정하기 위해 매우 중요하다. 인과관계 평가에는 다음과 같은 항목들이 고려되어야 한다.

가) 노출여부(exposure) :　실제로 임상시험용 의료기기를 적용하였는가?

나) 시간순서(time course) :　이상반응이 의료기기 적용 이후 적당한 시간적 순서에 따라 발생하였는가?

다) 개연성(likely) : 의료기기 적용이 기저질환이나 다른 요인보다 이상반응 발생을 더 잘 설명하는가?

라) 적용중지(dechaleenge) : 의료기기의 적용 중단으로 이상반응이 완화되거나 소실되었는가?

마) 재적용(rechallenge) : 의료기기의 재적용으로 이상반응이 재발하거나 약화되었는가?

이상반응의 인과관계를 평가한 후 평가결과를 구분하는 방법(세계보건기구 분류체계)은 다음과 같다.

| 분류 | 평가 결과 |
| --- | --- |
| 관련성이 명백함(Definitely) | 의료기기 적용과 타당한 시간관계를 보인다.  질환이나 다른 원인으로 설명될 수 없다.  적용중단 시에 반응이 있다.  재적용(해당하는 경우) 시에 반응을 보인다. |
| 관련성 많음(probable) | 의료기기 적용과 타당한 시간관계를 보인다.  질환이나 다른 원인으로 설명이 어렵다.  적용중단 시에 반응이 있다.  재적용이 불필요하다 |
| 관련성이 의심됨(possible) | 의료기기 적용과 타당한 시간관계를 보인다.  질환이나 다른 원인으로 설명할 수도 있다.  적용중단 시에 반응이 약하거나 불명확할 수 있다. |
| 관련성이 적음(unlikely) | 의료기기 적용과 시간 관계가 적을 것 같다.  질환이나 다른 원인으로 설명할 수도 있다.  적용중단 시에 반응이 약하거나 불명확할 수 있다. |
| 관련성이 없음(unrelated) | 의료기기 적용과 시간 관계가 없다.  질환이나 다른 원인이 분명하다.  적용중단 시에 반응이 없다. |
| 평가불가능(unclassified) | 정보가 불충분하거나 모순되어 평가가 불가능하다.  자료가 보충되거나 확인될 수 없다. |

이상반응과 시험용 의료기기와의 관계(또는 기타 원인 기저질환의 진행, 병용 치료 등)의 확실성의 정도는 아래의 관점에서 이상반응이 얼마나 잘 설명될 수 있는가에 따라 결정한다.

◆ 시험용 의료기기에 대해 알려진 작용

◆ 시험용 의료기기나 같은 계열의 의료기기에서 이전에 관찰되었던 유사한 작용

◆ 유사한 의료기기와 관련이 있다고 자주 보고된 반응들(예; 홍반, 혈액질환)

**16-5 이상반응의 추적 관찰**

임상시험 책임자 또는 담당자는 이상반응이 나타난 피험자에 대해 증상이 가라앉고 비정상적 임상 검사치가 기준치로 회복되거나, 혹은 관찰된 변화에 대해 만족스러운 설명이 될 때까지 추적 관찰하여야 한다.

**16-6 중대한 이상반응**

1) 중대한 이상반응의 정의

중대한 이상반응은, 사망, 생명에 위협을 주는 경우, 입원을 요하거나 기존의 입원기간을 연장시키는 경우, 지속적 또는 중요한 불구/무능한 상태를 초래하는 경우, 피험자 자손의 선천성 기형이나 장애를 초래하는 경우를 말한다. 이외에도 사망, 생명위협을 초래하지 않거나, 입원을 요하지 않는 중요한 의료 사건은 적절한 의학적인 판단을 근거로 하여, 그들이 환자 또는 피험자에게 위험을 주거나 또는 이 정의에 명시된 결과 중 한 가지를 방지하기 위하여 의료적 또는 수술적 조치가 요구될 때 중대한 이상반응으로 간주될 수 있다.

a)"생명을 위협하는 (Life Threatening)"의 정의

만일 환자가 이상반응이 발생하였을 때 즉시 사망의 위험에 처한다면 그 이상반응은 생명을 위협하는 것이다. 그러나 그 이상반응이 더 심각해 졌을 때 사망을 유발할 수 있는 이상반응은 포함되지 않는다.

b)"장애/불능 (Disability/Incapacitating)"의 정의

환자의 정상적인 생활을 수행하는 데에 실질적으로 또는 지속적으로 어려움을 유발한다면 이 이상반응은 환자가 일시적 또는 지속적인 장애로 고통 받는 것을 말한다.

c)"입원(Hospitalization)"의 정의

입원을 요하는 이상반응은 중대하다고 여겨진다. 임상시험 시작이후에 중증도나 빈도가 심화되지 않은 기존의 증상을 위해 이미 계획된 수술을 받기 위해서나 관례적인 임상절차상 입원을 한 경우에는 이상반응으로 분류되지 않는다.

이미 계획되어 있는 절차 중에 바람직하지 못한 증상이 발생한 경우에는 분류기준에 따라 중대한 또는 중대하지 않은 이상반응으로 보고되어야 한다. 일반적으로, 임상의의 진료실이나 외래에서 적절히 치료할 수 없는 관계로 피험자가 입원실이나 응급실에서 최소한 하룻밤 이상 체류하는 경우를 말한다. 입원으로 규정해야 할지가 모호한 경우나 입원이 필요한 임상적 상황이었는지의 여부가 명확하지 않을 경우에는 중대한 이상반응으로 간주한다.

d)"관례적인 임상절차 (Routine Clinical Procedure)"의 정의

임상시험 기간 중에 발생할 수 있으나 시험용 의료기기 접종이나 계획서에 명시된 절차의 진행을 방해하지 않는 임상상의 절차

e)기타

즉각적 생명의 위협을 주는 경우, 사망 또는 입원을 요하지는 않지만 피험자를 위태롭게 하거나 위의 정의에 열거된 결과가 나타나지 않도록 예방하기 위해 내과적 또는 외과적 중재가 요구되어지는 중요한 의학적 또는 과학적 판단을 이용하는데 익숙해져야 한다. 이러한 상황 또한 중대한 이상반응으로 간주된다.

2) 중대한 이상반응의 보고

가) 임상시험책임자 및 담당자는 다른 시험자, 심사위원회 및 처장에게 중대하고 예상하지 못한 모든 이상반응(이상의료기기반응, 심각한 이상반응/이상의료기기반응 포함한다)을 다음과 같은 기간 내에 “의료기기 시행규칙” 별지 제 35호 이상의료기기반응 신속보고서를 작성하여 가능한 신속히 보고 해야한다.

① 사망을 초래하거나 생명을 위협하는 경우에는 의뢰자가 이 사실을 보고받거나 알게 된 날로부터 7일 이내. 이 경우 상세한 정보를 최초 보고일로부터 8일 이내에 추가로 보고하여야 한다.

② 그 밖의 중대하고 예상하지 못한 모든 이상반응이 나타난 경우에는 임상시험책임자 및 담당자가 이 사실을 보고받거나 알게 된 날로부터 15일 이내

나) 임상시험책임자 및 담당자는 가)항의 보고와 관련하여 추가적인 안전성 정보를 주기적으로 해당 이상반응이 종결(해당 이상반응의 소실 또는 추적조사의 불가 등)될 때까지 보고하여야 한다.

다) 임상시험책임자는 처장에게 제1항의 규정에 의한 이상반응을 보고하고자 하는 경우에는 식품의약품안전처 “의료기기 시행규칙” 별지 제36호 서식에 의한 이상반응보고서에 ‘3) 안전성 관련 사항의 보고’ 규정에 의하여 보고받은 내용을 첨부하여 제출하여야 한다.

**16-7 안전성과 관련한 보고**

가) 시험책임자는 모든 중대한 이상반응(임상시험계획서나 임상시험자 자료집 등에서 즉시 보고하지 않아도 된다고 명기한 것을 제외한다)을 임상시험계획서에 정한 기간 내에 “의료기기법 시행규칙“의 서식 제 35호 서식에 따른 이상의료기기반응 신속보고서로 식약처나 심사위원회에게 보고하여야 한다. 이 경우 시험책임자는 피험자의 신상에 관한 비밀을 보호하기 위하여 피험자의 성명, 주민등록번호 및 주소 등 피험자의 신상에 관한 비밀을 보호하기 위하여 피험자의 성명, 주민등록번호 및 주소 등 피험자식별코드를 사용하여야 하며, 이상반응의 보고에 관한 관련 지침이 있는 경우에는 이에 따라야 한다.

나) 시험책임자는 임상시험계획서에서 안전성 평가와 관련하여 별도로 정한 이상반응이나 실험실실험 결과의 이상 등에 대하여 임상시험계획서에서 정한 기간 내에 임상시험계획서에서 정한 보고방법에 따라 식약처나 심사위원회에게 보고하여야 한다.

다) 사망 예를 보고하는 경우 시험책임자는 심사위원회에게 부검보고서(부검을 실시한 경우에 한한다)와 사망진단서 등의 추가정보를 제공하여야 한다.

◆시험책임자의 의무

가) 시험책임자는 임상시험용 의료기기의 안전성에 대한 평가를 지속적으로 실시하여야 한다.

나) 시험책임자는 피험자의 안전을 위협하거나, 임상시험의 실시여부에 영향을 미치거나 심사위원회의 결정사항을 변경해야 할 만한 임상시험용 의료기기의 안전성에 관한 정보를 취득한 경우에는 시험자 및 식품의약품안전처장에게 이를 신속히 보고하여야 한다.

**16-8 이상 의료기기 반응**

1) 이상 의료기기 반응의 정의

임상시험자자료집 또는 의료기기의 첨부문서 등 이용 가능한 의료기기 관련 정보에 비추어 이상의료기기반응의 양상이나 위해의 정도에서 차이가 나는 것을 말한다.

2) 이상 의료기기 반응의 보고

가) 임상시험책임자 혹은 임상연구자는 심사위원회(시험책임자가 심사위원회에 보고하지 않았거나 보고한 사항을 변경할 필요가 있는 경우에만 해당한다) 및 식품의약품안전처장에게 중대하고 예상하지 못한 모든 이상의료기기반응을 다음의 구분에 따른 기간 내에 보고하여야 한다.

나) 임상시험책임자 혹은 임상연구자는 가)에 따라 보고한 이상의료기기반응의 추가적인 정보가 있는 경우에는 해당 이상의료기기반응이 종결(해당 이상의료기기반응이 사라지거나 추적조사가 불가능하게 되는 것을 말한다)될 때까지 보고하여야 한다.

다) 임상시험책임자가 식품의약품안전처장에게 가)에 따라 이상의료기기반응을 보고하고자 하는 경우에는 별지 제 36호 서식에 따른 이상의료기기반응 보고서에 제 7호 과목 1)에 따라 시험책임자가 보고한 별지 제 35호 서식에 따른 이상의료기기반응 신속보고서를 첨부하여 제출하여야한다.

**16-9 임신**

임상시험 실시 중 또는 종료 후에 임신 사실을 알게 된 피험자는 임신의 시기가 시험용 의료기기 투여기간 중이었는지, 시험용 의료기기의 마지막 적용 30일 이내이었는지를 연구자에게 알려야 한다. 담당 연구자는 임상시험책임자에게 24시간 이내에 중대한 이상반응 보고서 양식을 작성하여 보고해야 한다. 이 때 임신에 대한 최초 보고에 대한 기록을 제공하여야 한다. 모든 임신은 추적관찰 되어야 하며 윤리적인 목적으로만 수집하여야 한다.

**17. 그 밖에 임상시험을 안전하고 과학적으로 실시하기 위하여 필요한 사항**

1) 증례기록서(Clinical Report Form, CRF)

증례기록서에는 개개 피험자별로 임상시험계획서에서 요구한 정보를 기록한다. (증례기록서 별도 첨부)

2) 시험책임자 이력서

(CV 별도 첨부)

3) 임상시험용 의료기기의 사용 및 관리

임상시험용 의료기기는 해당 임상시험실시기관의 장이 지정한 자가 관리한다. 임상시험용 의료기기는 기재사항에 기술되어 있는 대로 취급, 저장하며 “임상시험용”이라는 문구가 있어야 한다.

4) 임상시험에 들어가기 전에 피험자에게 시험내용 및 시험제품의 효과·이상반응 및 안전성에 대한 모든 사항을 설명한 후 피험자 자신이 자발적으로 본 임상시험에 참여하겠다는 동의서를 받고 임상시험에 들어간다. 피험자 또는 법정대리인이 동의서 서식, 임상시험 대상자 설명서 및 기타 문서화된 정보를 읽을 수 없는 경우에는 공정한 입회자가 동의를 얻는 전 과정에 참석한다. 이 경우, 동의서 서식, 임상시험 대상자 설명서 및 기타 문서화된 정보를 피험자 또는 법정대리인에게 읽어주고 설명한 후, 피험자 또는 법정대리인이 피험자의 임상시험 참여를 구두로 동의하고 가능하다면 동의서 서식에 서명하고 자필로 해당 날짜를 기재하게 한 다음, 공정한 입회자도 동의서 서식에 서명하기 전에 동의서 서식과 임상시험 대상자 설명서 및 기타 문서화된 정보가 정확하게 피험자나 대리인에게 설명되었고, 이들이 해당사실을 이해하였다는 것과 동의를 얻는 과정이 피험자나 법정대리인의 자유의사에 따라 진행되었다는 것을 확인해야 한다. (피험자 설명서 및 동의서 서식 별도 첨부)

**18. 피험자 보상에 대한 규약**

본 피험자 보상에 대한 규약은 현 임상시험에 참여하여 임상시험에 사용되는 피험자에 대하여 적용되는 보상규약입니다.

**18-1 피험자 보상 사유**

의료기기 임상시험 관리기준에 따라 이루어진 임상시험에 있어서 임상 의료기기로 인하여 피험자에게 발생한 유해하고 의도되지 않은 반응에 의한 피험자의 신체상의 손상에 대하여, 다음의 경우 본 보상규약 및 관련 법률의 규정에 따라 이를 보상합니다.

1) 일시적 통증 또는 쉽게 치료될 수 있는 정도의 손상으로서 시험기관이 이에 대한 치료가 필요하다고 판단되는 경우(보상범위는 필요한 치료비에 한정함)

2) 입원 또는 입원 기간의 연장이 필요한 경우

3) 지속적 또는 의미 있는 불구나 기능 저하를 초래하는 경우

4) 선천적 기형 또는 이상을 초래하는 경우

5) 사망을 초래하거나 생명을 위협하는 경우

**18-2 보상요건**

본 보상규약에 따른 피험자 보상은 다음의 요건 하에 이루어집니다.

1) 본 임상시험용 의료기기로 인하여 발생한 신체상의 손상일 것

2) 시험자가 식품의약품안전청장의 승인을 받은 임상시험계획서의 제반내용을 준수하였을 것

3) 시험자의 명백한 과실이나 의무태만에 기인하지 아니하였을 것

4) 피험자가 시험책임자 또는 시험담당자의 제반 지시사항을 모두 준수하였을 것

5) 피험자가 당해 신체상의 손상으로 인한 손해의 발생을 최소화하기 위하여 조치를 취하였을 것

**18-3 보상 제외사유**

전 2조의 규정에 불구하고, 다음의 각 경우에는 본 보상규약에 따른 보상범위에서 제외됩니다.

1) 시험용 의료기기 적응증에 대한 효과, 효능의 불충분으로 인한 손상

4) 피험자의 부주의에서 초래된 손상

**18-4 보상평가기준**

1) 예상된 의료기기 이상반응에 대하여 당사자들 간에 미리 합의한 보상액 또는 조치가 있는 경우, 당해 기준에 따라 이를 보상합니다.

2) 그 외의 경우에는 신체손상의 정도, 성격, 지속기간, 유사사례 등을 종합적으로 고려하여 당사자들 간에 합의한 보상방법에 따라 이를 보상합니다.

2) 당사자들 간에 전항의 합의가 이루어지지 아니한 경우에는, 법원의 판결 및 이에 준하는 결정의 확정내용에 따라 보상합니다.

**18-5 보상절차**

본 보상규약에 따른 신체상의 손상을 입은 피험자는 임상시험의 시험책임자나 시험기관에 필요한 의료조치를 요청하여야 합니다. 처치 후 이의를 통보하지 아니한 경우, 양 당사자는 위 통보내용에 따른 보상에 합의한 것으로 양해합니다. 이에 이의를 통보한 경우 피험자에게 보상대상 해당여부 및 보상기준에 관하여 판단할 객관적인 전문가를 복수로 추천하고, 피험자를 추천일로부터 3일내에 추천인 중 1명을 지명합니다. (피험자가 지명하지 않을 경우 의뢰자가 임의 택일합니다.)

**18-6 적용범위**

1) 본 보상규약은 위 임상시험에 참여하는 피험자에 대하여 다른 약정이 없는 한 그 범위 내에서 일반적으로 적용됩니다.

2) 피험자가 임상시험에 관한 보상에 대하여 시험책임자 및 연구자의 승인을 받지 아니하고 임상시험과 관련된 다른 제 3자와 체결한 일체의 합의내용은 의뢰기관에 대하여 효력이 없습니다.

앞에서 언급한 여러 제반 내용을 참고하여, 환자가 본 연구에 의해 어떠한 불이익이라도 받지 않도록 주의하며, 만약 본 연구에 의해 피해가 발생한 경우 위의 규약에 의거하여 책임을 질 것을 서약합니다.

2015년 월 일

서울아산병원 유방암내분비외과 손병호 (인)

**19. 임상시험 후 피험자의 진료에 관한 사항**

임상시험에서 탈락되거나 반응이 없는 피험자는 다른 적절한 치료를 받을 수 있도록 지도하며, 임상시험이 종료된 피험자 중 치료효과가 나타나지 않은 피험자는 다른 치료법으로 전환한다. 다만, 임상시험 도중이나 종료 후 시험제품에 의한 중대한 이상반응이 발생하거나 질병이 악화되는 경우 언제든지 시험자 또는 시험담당자의 지시에 따라 진료를 받을 수 있으며 피해자 보상 규약에 의거하여 보상받을 수 있다.

1. 원칙

1) 환자의 신체적인 손상(사망포함)에 대해 진료 및 보상한다.

2) 손상의 원인이 임상시험용 의료기기에 의해 발생했을 때 환자에게 진료 및 보상한다.

3) 지속적으로 불구가 될 수 있을 정도의 보다 심각한 손상에 대해서만 진료 및 보상한다.

4) 임상시험용 의료기기에 의해 생긴 부작용이나 부작용 처치 과정에서 발생된 손상이 있는 경우, 시험용 의료기기가 직접적인 원인이 된 손상에 대하여 진료 및 보상한다.

2. 다음 경우에는 진료 또는 보상하지 아니한다.

1) 임상시험용 의료기기인 Guardix-SG가 아닌 다른 의료기기로 발생한 부작용에 의한 손상

2) 시험용 의료기기 적응증에 대한 효과 또는 혜택을 제공하지 못한데 대한 보상

3) 서로 합의한 프로토콜에서 이탈함으로(또는 임상시험자의 지시에 따르지 않음으로) 야기된 손상

4) 피험자의 부주의에서 초래된 손상

3. 보상평가기준

1) 보상수준은 손상의 본질, 그 정도, 지속성 여부 등에 적절한 액수여야하며, 한국의 법정에 의해 유사 손상들에 대해 일반적으로 지급되는 것과 동일해야 한다.

2) 보상수준에 대해서 이견이 있을 경우, 양자가 수용할 수 있는 전문가의 자문을 구해야 한다.

4. 보상절차

1) 보상청구서 접수 - 임상시험 대상자 설명서에 나와 있는 시험자에게 연락하여 접수

2) 보상대상 심사 - 상기한 내용과 비교하여 심사

3) 보상수준 심사(자문의) 및 실사(보험사) - 보상 금액 결정 (보험에 가입된 상태로 임상 진행)

4) 보상금 입금 조치 - 피험자 또는 대리인

앞에서 언급한 여러 제반 내용을 참고하여, 환자가 본 연구에 의해 어떠한 불이익이라도 받지 않도록 주의하며, 만약 본 연구에 의해 피해가 발생한 경우 위의 규약에 의거하여 책임을 질 것입니다.

**20. 피험자의 안전보호에 관한 대책**

**20-1 임상시험 실시기관**

임상시험실시기관의 장은 임상시험의 실시에 필요한 임상실험실, 설비와 전문인력을 갖추어야 하고, 긴급 시 필요한 조치를 취할 수 있도록 하는 등 해당 임상시험을 적절하게 실시할 수 있도록 하여야 한다. 피험자의 안전보호를 위해 임상시험을 조기종료 또는 일시중지 시켰을 경우, 시험기관의 장은 이 사실을 식품의약품안전처장에게 즉시 알리고, 조기종료 및 일시중지에 대한 상세한 사유서를 제출하여야 한다.

**20-2 임상시험심사위원회**

임상시험심사위원회(Institutional Review Board)는 국내 법규/관례에 따라 구성되어 있어야 한다. IRB는 피험자의 권리, 안전, 복지를 보호해야 하며, 취약한 환경에 있는 피험자가 임상시험에 참여하는 경우에는 그 이유의 타당성을 면밀히 검토하여야 한다.

임상시험심사위원회는 임무를 수행함에 있어 피험자의 시험참가 동의가 적절하게 얻어지지 않았거나 임상시험이 임상시험계획서에 따라 진행되지 않은 경우 또는 심각한 이상반응/이상의료기기반응이 나타난 경우에는 임상시험 일부 또는 전부에 대하여 중지 명령을 할 수 있다. 피험자가 임상시험에 참여하는 대가로 금전적 보상을 받는 경우 심사위원회는 그 보상액, 보상 방법 및 금전적 보상이 피험자의 임상시험 참여에 부당한 영향을 미치는지 여부를 검토하여야 하며, 이 경우 금전적 보상이 피험자의 임상시험 참여 정도와 참여 기간에 비추어 적절한지 여부 및 피험자에 대한 보상이 임상시험에 끝까지 참여할 것을 조건으로 하는지 여부를 검토하여야 한다. 또한 임상시험을 종료하지 못한 피험자에 대한 보상 방안 유무를 확인하여야 한다.

**20-3 시험자**

시험자(Investigator)라 함은 시험책임자, 시험담당자를 말한다. 현재 위 연구는 연구자 주도 임상시험으로 연구자는 임상시험심사위원회 및 식품의약품안전처장의 승인을 득한 임상시험계획서를 준수하여 임상시험을 실시하여야 한다.

피험자 모집 시 임상시험내용을 충분히 숙지하고 임상시험 전에 모든 피험자에게 상세히 설명하여 이해할 수 있도록 하고, 피험자의 자발적인 동의를 구한다. 임상시험 중 또는 임상시험 이후에도, 시험자는 임상적으로 의미 있는 실험실적 검사치의 이상을 포함하여 임상시험에서 발생한 모든 이상반응에 대해 피험자가 적절한 의학적 처치를 받을 수 있도록 조치하여야 하고, 시험자가 알게 된 피험자의 병발질환에 대해 의학적 처치가 필요한 경우 이를 피험자에게 알려주어야 한다.

시험자는 피험자의 보상 등에 관련된 책임을 가지며 임상시험계획서의 절차에 따라 이루어지도록 하여야 하며, 대상 피험자의 문제점을 적극적으로 대응한다. (책임연구자 CV 첨부)

**20-4 임상시험계획서의 승인**

임상시험을 시작하기 전에 국내법규에 따라 임상시험계획서 및 관련 서류를 임상시험심사위원회에 제출한다. 임상시험 의뢰자와 시험자는 최초의 피험자를 등록시키기 전에 모든 윤리적․법적 요건이 충족되었다는 것을 문서로 상호 간에 통보해 주어야 한다. 임상시험기관의 승인 후 계약을 체결한다. (임상시험 계약서 별도 첨부)

**20-5 비밀보장**

모든 피험자명은 비밀을 유지하고 시험 도중 부여한 번호에 의해 기록 및 평가 시 피험자를 확인한다. 피험자에게 모든 시험 자료가 컴퓨터에 저장되고 엄격히 비밀사항으로 다루어진다는 것을 알려준다. 서명을 받은 피험자동의서는 시험자가 보관한다. 본 계획서에 서명함으로써 시험자는 임상시험에 참가하는 피험자로부터 올바르게 동의서를 획득하기로 동의한 것이며 요청이 있을 경우 실사를 받는 데에도 동의한 것이다. 시험자는 피험자번호 및 피험자명이 기록된 리스트를 갖추어 놓아 나중에 기록을 찾을 수 있도록 한다. 피험자 동의서와 피험자 리스트는 3년간 보관한다.

**20-6 시험결과의 기록 및 이용**

본 임상시험 중에 수집된 모든 자료는 증례기록서에 시험자가 기록하고 시험자는 증례기록서의 원본을 보관해야 한다.

증례기록서를 기입할 때에는 흑색볼펜(연필이나 만년필은 사용하지 말 것)으로 분명하고 알아볼 수 있도록 기록해야 하며 자료가 누락된 경우에는 시험자가 타당한 설명을 붙여야 한다.

증례기록서에 기입한 내용을 수정하는 경우에는 원기록을 알아볼 수 있도록 해야 하고 수정한 시험담당자의 서명을 해야 한다. (증례기록서 별도 첨부)

**20-7 기타**

본 시험계획은 Helsinki 선언에 입각하여 피험자의 권리와 복지를 염두에 두고 준비된 것으로, 임상시험 책임자 또는 담당자는 본 시험의 목적 및 모든 가능성에 대하여 설명하고. 피험자 자신이 자발적으로 임상시험에 참여하겠다는 피험자 동의서에 서명 날인한 피험자를 대상으로 한다. (헬싱키선언 별도 첨부)

임상시험 담당자 및 참여 연구진은 연구계획을 정확히 분석, 숙지토록 하며 시험 책임자는 예기치 않은 이상반응 등의 출현에 대한 충분한 대처와 필요에 따른 보고, 시험참여 연구진에 대한 충분한 교육 등 사전 조치를 취하며 임상시험의 진행은 ‘의료기기 임상시험 실시 기준’에 합당하게 진행된다.

**21. 참고문헌**

1. Kootstra JJ, Weebers J, Rietman JS, Vries JV, Baas PC, Geertzen JH, Hoekstra HJ (2010) A longitudinal comparison of arm morbidity in stage I-II breast cancer patients treated with sentinel lymph node biopsy, sentinel lymph node biopsy followed by completion lymph node dissection, or axillary lymph node dissection. Ann Surg Oncol 17:2384-2394

2. Hayes SC, Rye S, Battistutta D, DiSipio T, Newman B (2010) Upper-body morbidity following breast cancer treatment is common, may persist longer-term and adversely influences quality of life. Health Qual Life Outcomes 8:92

3. Kwan W, Jackson J, Weir LM, Dingee C, McGregor G, Olivotto IA (2002) Chronic arm morbidity after curative breast cancer treatment: prevalence and impact on quality of life. J Clin Oncol 20:4242–4248

4. Yang EJ, Park WB, Seo KS, Kim SW, Heo CY, Lim JY (2010) Longitudinal change of treatment-related upper limb dysfunction and its impact on late dysfunction in breast cancer survivors: a prospective cohort study. J Surg Oncol 101:84–91

5. Cheville AL, Tchou J (2007) Barriers to rehabilitation following surgery for primary breast cancer. J Surg Oncol 95:409–418

6. Stubblefield MD, Custodio CM (2006) Upper-extremity pain disorders in breast cancer. Arch Phys Med Rehabil 87:S96–S99 (quiz S100–S101)

7. Karki A, Simonen R, Malkia E, Selfe J (2005) Impairments, activity limitations and participation restrictions 6 and 12 months after breast cancer operation. J Rehabil Med 37:180–188

8. Lee SA, Kang JY, Kim YD, An AR, Kim SW, Kim YS, Lim JY (2010) Effects of a scapula-oriented shoulder exercise programme on upper limb dysfunction in breast cancer survivors: a randomized controlled pilot trial. Clin Rehabil 24:600–613

9. Shamley D, Srinaganathan R, Oskrochi R, Lascurain-Aguirrebena I, Sugden E (2009) Three-dimensional scapulothoracic motion following treatment for breast cancer. Breast Cancer Res Treat 118:315–322

10. Caban ME, Freeman JL, Zhang DD, Jansen C, Ostir G, Hatch SS, Goodwin JS (2006) The relationship between depressive symptoms and shoulder mobility among older women: assessment at one year after breast cancer diagnosis. Clin Rehabil 20:513–522

11. Agrawal A, Ayantunde AA, Cheung KL (2006) Concepts of seroma formation and prevention in breast cancer surgery. ANZ J Surg 76:1088–1095

12. Lotze MT, Duncan MA, Gerber LH, Woltering EA, Rosenberg SA (1981) Early versus delayed shoulder motion following axillary dissection: a randomized prospective study. Ann Surg 193:288–295

13. Shamley DR, Barker K, Simonite V, Beardshaw A (2005) Delayed versus immediate exercises following surgery for breast cancer: a systematic review. Breast Cancer Res Treat 90:263–271

14. Mais V, Bracco GL, Litta P, Gargiulo T, Melis GB (2006) Reduction of postoperative adhesions with an auto-crosslinked hyaluronan gel in gynaecological laparoscopic surgery: a blinded, controlled, randomized, multicentre study. Hum Reprod 21:1248–1254

15. Kim JH, Lee JH, Yoon JH, Chang JH, Bae JH, Kim KS (2007) Antiadhesive effect of the mixed solution of sodium hyaluronate and sodium carboxymethylcellulose after endoscopic sinus surgery. Am J Rhinol 21:95–99

16. Park JS, Cha SJ, Kim BG, Choi YS, Kwon GY, Kang H, An SS (2011) An assessment of the effects of a hyaluronan-based solution on reduction of postsurgical adhesion formation in rats: a comparative study of hyaluronan-based solution and two film barriers. J Surg Res 168:49–55

17. Yang EJ, Kang E, Jang JY, Kim D, Yom CK, Lim JY, Kim SW (2012) Effect of a mixed solution of sodium hyaluronate and carboxymethyl cellulose on upper limb dysfunction after total mastectomy: a double-blind, randomized clinical trial. Breast Cancer Res Treat. 136:187-194.

18. Falk K, Björquist P, Strömqvist M, Holmdahl L (2001) Reduction of experimental adhesion formation by inhibition of plasminogen activator inhibitor type 1. Br J Surg. 88:286-289.

19. Burns JW, Colt MJ, Burgees LS, Skinner KC (1997) Preclinical evaluation of Seprafilm bioresorbable membrane. Eur J Surg Suppl. 577:40-48.

20. Kwon SW, Lim SH, Lee YW, Lee YG, Chu BY, Lee JH, Lee YM (2006) Anti-adhesive Effect of Poloxamer/Alginate/CaCl2 Mixture in the Rat Model. J Korean Surg Soc. 71:280-287

21. Kong CG, In Y, Cho HM, Suhl KH (2011) The effects of applying adhesion prevention gel on the range of motion and pain after TKA. Knee. 18:104-107

22. Park JH, Jeong JJ, Kang SW, Nam KH, Hang Chang HS, Chung WY, Park CS (2006) The Efficacy and Safety of Guardix-SG^Ⓡ^ in Patients Who Are Undergoing Thyroid Surgery: A Randomized, Prospective, Double-blinded Study. Korean Journal of Endocrine Surgery 9:127-132

23. de Rezende LF, Franco RL, de Rezende MF, Beletti PO, Morais SS, Gurgel MS (2006) Two exercise schemes in postoperative breast cancer: comparison of effects on shoulder movement and lymphatic disturbance. Tumori. 92:55-61

24. Box RC, Reul-Hirche HM, Bullock-Saxton JE, Furnival CM (2002) Shoulder movement after breast cancer surgery: results of a randomised controlled study of postoperative physiotherapy. Breast Cancer Res Treat. 75:35-50.

25. M. M. F., Oliveira; M. S. C., Gurgel; M. S., Miranda; M. A., Okubo; L. F. A., Feijó; G. A., Souza (2009) Efficacy of shoulder exercises on locoregional complications in women undergoing radiotherapy for breast cancer: clinical trial. Brazilian Journal of Physical Therapy 13:136-143

26. Lim JY, Lee HY, Song JH, Kang JW, Lee JY (2005) Evaluation of the Reliability, Construct Validity, and Responsiveness of the Korean Version of the DASH. The Joumal of the Korean Society for Surgery of the Hand. 10:192-198

27. D. Gould et al. (2001) Visual Analogue Scale(VAS). 706

28. Chow SC, Shao J, Wang H (2008) Sample size calculations in clinical research(second edition). Taylor & Francis Group, LLC.
