# Supplementary material for: The effects of poloxamer and sodium alginate mixture (Guardix-SG®) on range of motion after axillary lymph node dissection: A single-center, prospective, randomized, double-blind pilot study
Source: PLoS One. 2020 Sep 23;15(9):e0238284. doi: 10.1371/journal.pone.0238284 (PMC7510996; doi:10.1371/journal.pone.0238284)
Supplement: S3 File — (DOCX) [file pone.0238284.s005.docx]

**Inclusion/Exclusion Criteria, and Calculation of Subject Number**

**1 Inclusion Criteria**

(1) The adult over 20 years old

(2) Patient who is diagnosed as a breast cancer

(3) Breast cancer patient who underwent the modified radical mastectomy or breast preservation surgery and the axillary lymph node dissection(Including the patient who received the preoperative chemotherapy after undergoing the axillary lymph node dissection)

**2 Exclusion Criteria**

(1) Intraepithelial carcinoma

(2) Stage IV

(3) The patient who underwent the sentinel node biopsy only

(4) The patient who underwent the total mastectomy and the simultaneous reconstruction surgery

(5) When the shoulder movement disorder derived from the other preoperative shoulder-related diseases exists.

(6) The patient with a breast cancer on the east side or the history of surgery on the axillaries

(7) The patient who is pregnant or breastfeeding

**3 The target enrollment number of subject and the grounds of estimation [28]**

This clinical trial is to evaluate the efficacy of Guardix SG, anti-adhesive agent composed of alginate and poloxamer in the range of motion of patient’s shoulder joint who is scheduled to undergo the modified radical mastectomy or breast preservation surgery and the axillary lymph node dissection.

According to the related literature, over 10 degree-difference in the ROM considered to be significant clinically [17] and over 10 degree-difference between the experimental group and the control in the ROM is considered to be significant. Therefore, we estimate the 192 persons(total) as a target enrollment number by assigning 96 subjects for each group in consideration of the drop-out rate of 10% under the precision degree of 0.025, power of 90%, and standard deviation of 10 [25] using Pass software.

* The rationale for our study is as follows.

**H_0_ : D ≤ 10 H_1_ : D〉10 (D=Experimental group(EG) ROM Mean – Control group(CG) ROM Mean)**

* Basis of Calculation: Assumptions for estimating the effective subject number.

① Significance level α = 0.025

② The EG estimation number is 1 time that of CG.

③ Type 2 error (β) is 0.1, and statistical power 90% is maintained.

④ The difference in the average value between two groups: 10 degree.


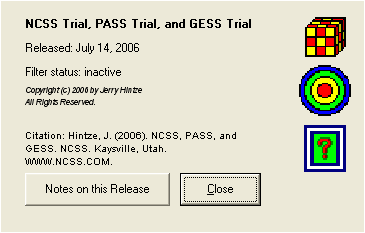


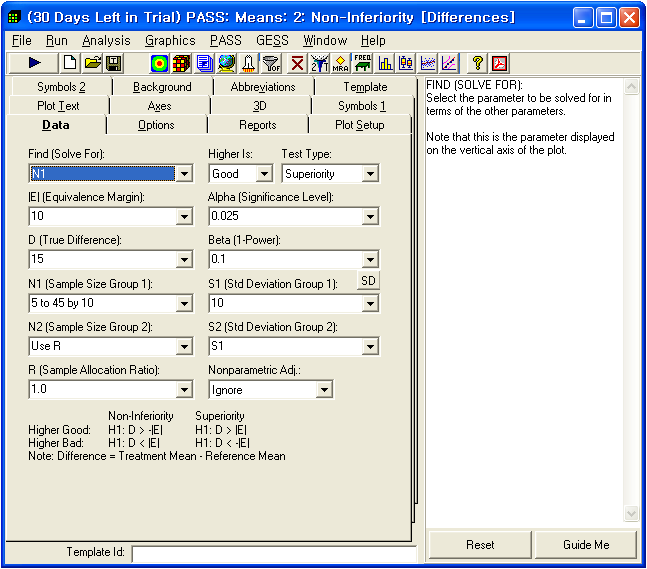


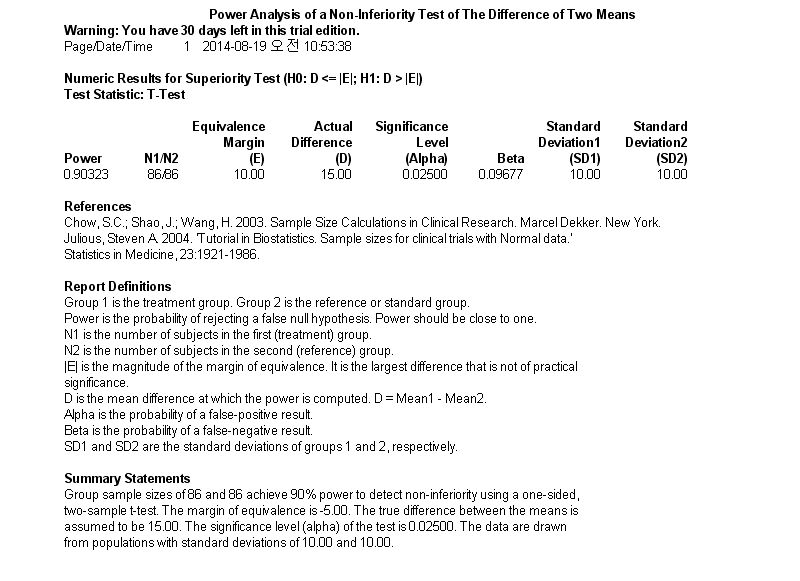


| Power | N1/N2 | Equivalence Margin  (E) | Actual  Difference  (D) | Significance  Level  (Alpha) | Beta | Standard  Deviation1  (SD1) | Standard  Deviation2  (SD2) |
| --- | --- | --- | --- | --- | --- | --- | --- |
| 0.90022 | 23/23 | 5 | 10 | 0.025 | 0.09978 | 5 | 5 |
| 0.80044 | 17/17 | 5 | 10 | 0.025 | 0.19956 | 5 | 5 |
| 0.90004 | 86/86 | 5 | 10 | 0.025 | 0.09996 | 10 | 10 |
| 0.80003 | 64/64 | 5 | 10 | 0.025 | 0.19997 | 10 | 10 |
| 0.90022 | 191/191 | 5 | 10 | 0.025 | 0.09978 | 15 | 15 |
| 0.80044 | 143/143 | 5 | 10 | 0.025 | 0.19956 | 15 | 15 |
| 0.91250 | 23/23 | 10 | 15 | 0.025 | 0.08750 | 5 | 5 |
| 0.80704 | 17/17 | 10 | 15 | 0.025 | 0.19296 | 5 | 5 |
| 0.90323 | 86/86 | 10 | 15 | 0.025 | 0.09677 | 10 | 10 |
| 0.80146 | 64/64 | 10 | 15 | 0.025 | 0.19854 | 10 | 10 |
| 0.90022 | 191/191 | 10 | 15 | 0.025 | 0.09978 | 15 | 15 |
| 0.80044 | 143/143 | 10 | 15 | 0.025 | 0.19956 | 15 | 15 |

Suppose that the 10% subject is dropped out in the middle of trial, the necessary total subject is 192 persons composing of 96 persons for the experimental group and 96 persons for the control.

| Target subject number | EG | CG | In total subject number |
| --- | --- | --- | --- |
| Subject number for final efficacy evaluation | 86 | 86 | 172 |
| Subject number in consideration of drop-out rate (10%) | 96 | 96 | 192 |

**9. Period of Clinical Trial**

30 months subsequent to the approval date from IRB of MFDS

* Subject enrollment period: 12 months

* Period for observation study and clinical trial: 12 months

* Statistic processing period: 4 months

* Result report preparation period: 2 months

**10. Methods of Clinical Trial**

**10.1 Investigational device**

(1) Investigational device

1) Product-License No.: 09-826 (Code: BM2102JO)

2) Product name: Deep-cavity wound dressing

3) Model name: GUARDIX SG

4) General name: Anti-adhesion agent.(solution)

(2) Raw materials

Poloxamer, Sodium alginate

(3) Shape·structure and measurements

1) Shape·structure

GUARDIX SG solution is a sterilized non-febrile, colorless, transparent viscous poloxamer/sodium alginate mixture solution. It provides a temporary viscous/lubricative coating on the wound surface after surgery. It is contained in a pre-filled syringe, and packed under a sterilized environment for use.


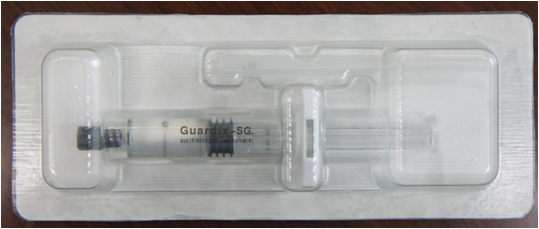

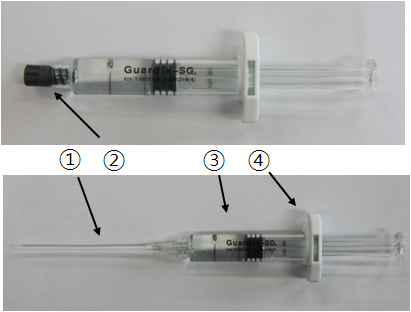
A) Picture of exterior

B) Explanation of exterior

① Catheter tube: Guardix SG to flow toward the surface of patient’s wound subsequent to the surgery.

② Stopper: To seal the syringe in order for the liquid not to leak.

③ Syringe: To contain the liquid of Guardix SG.

④ Finger grip: To grip the finger

2) Size and weight

A) Exterior size

The solution of Guardix SG (3ml and 6ml) is pre-filled within the syringe(material: cycloolefine copolymer, capacity: 5ml and 10ml) and the syringe’s tip closed with the lid is packaged within the container which has a polyester fixer. The catheter (material: Polyethylene terephthalate (Teflon)) which guides the injected liquid of solution is packaged with the Tyvek connected to the package.

B) Weight: 3ml, 6ml

**10.2 Comparator device**

The Comparator device referred as an inactive control isn’t used in this clinical trial.

[Currently, it is uncommon to apply the anti-adhesive agent onto the surgical site of patient who underwent the modified radical mastectomy or breast preservation surgery and the axillary lymph node dissection and it is safe to apply nothing to the surgical site. Therefore, we couldn’t find the reference medical device and didn’t assigned any medical device as the control.]

**10.3 Methods of Clinical Trial**

(1) Clinical Trial Design

The eligibility evaluation is conducted with the subject who underwent the modified radical mastectomy or breast preservation surgery and the axillary lymph node dissection at Asan medical center and signed at the consent form voluntarily subsequent to the screening test. The subject who isn’t included in the exclusion criteria visits the hospital on the operation day and undergoes the modified radical mastectomy or breast preservation surgery and the axillary lymph node dissection. Then, the subject is assigned to the investigational group or control according to the random assignment slip.

 EG: Guardix SG

 CG: Not applicable

That means the investigational device will be or won’t be applied onto the surgical site of subject depending on her group. Since the investigational device is applied inside of subject in the middle of surgery under anesthetic, the subject is unaware of treatment’s detail.

The related tests will be performed on the 7th, 3, 6 and 12 months subsequent to the surgery. In addition, the double-blind policy is maintained since the application of investigational device is performed by the expert who has a medical expertise and technique on the surgery and the application of investigational device, and the evaluator couldn’t know about the rule of random assignment and the allocation of subject to the group since the evaluator isn’t involved to the application of investigational device.

(2) Random Assignment of Subjects

In this clinical trial, random assignment was used to reduce bias that could be involved in the assignment of EG and CG, and to enhance the effects of comparison. To compare the precise difference in the range of motion of patient who underwent the partial mastectomy or total mastectomy, the subjects are assigned to each group randomly in similar proportion.

Our random assignment method is the block random assignment performed after generating random numbers (upto 4 places of decimals) for each subject using Excel’s random function (RAND). The sealed assignment slip is distributed to the subject. The sponsor of clinical trial provides the blinded slip which the investigational group or control is written down according to the randomly assigned subject table.

Subject numbers were given by each clinical trial institution, and the institution assigned a screening number to each subject who signed the written consent and completed the screening test. Upon completion of the screening process, and if a subject was recognized to be appropriate for this study, a random assignment number was given in sequence, then treated with the investigational device.

The screening number as well as the random assignment number of a dropout subject was also dropped out. A new subject must be provided with a new subject screening number and random assignment number.

(3) Code Breaking

The blindness must not be disclosed during the clinical trial period in any case. The blindness can only be disclosed if the subjects are in emergency situation, and the group they belong to is absolutely required to be identified. In this case, the blindness can be disclosed according to the appropriate process, and details must be recorded in written documents.

(4) The method of surgery and the application method of medical device

Subsequent to surgery, the 3ml(injection volume) of Guardix SG is applied to the subject who underwent the modified radical mastectomy or breast preservation surgery and the axillary lymph node dissection, the standard surgery method to treat the breast cancer.

**11. Monitoring Items and Monitoring Methods**

**11-1 Monitoring Items and Methods**

(1) Information Consent and Demographical Data

Before participating in the clinical trial, the purpose and procedure of clinical trial are explained to subject in detail and demographical information such as the receipt of the written consent and date, subjects’ initials, height, and BMI is recorded in the CRF. The copy of informed consent form is provided to the subject after receiving the written consent from subject.

(2) Past History and Physical function

The subject will be informed about the clinical trial continually. We recorded the stage of disease, medical history, the info related to the predisposing conditions on the shoulder joint, blood test, the range of shoulder joint’s motion, the evaluation on the exercise capability of upper limbs, and the evaluation on pain derived from the movement.

(3) Subject Eligibility Evaluation (Inclusion/Exclusion Criteria checking)

recorded the result conducted after evaluating whether the suitable subject is selected according to the inclusion/exclusion criteria based on the consent for the participation of clinical trial, demographical survey, medical history, the preoperative histological diagnosis, and etc.

(4) Observation items related to the surgery

- Basic information on the surgery

Operation date, Name of surgery, histopathological diagnosis, TNM stage, postoperative therapy (cancer chemotherapy, anti-hormone therapy, target therapy, radiotherapy)

- Safety evaluation

Adverse events (complication discovered during the hospitalization and surgery-related complication), Measurement of drainage volume subsequent to surgery, Observation of infectious symptom, Complete blood count exam (POD#7)

(5) Physical function (Before surgery, after surgery 3, 6, 12 months)

- Efficacy evaluation

Shoulder range of motion (Abduction, Horizontal abduction, Flexion, External rotation), Evaluation on the upper limbs’ locomotive capacity, the evaluation on pain derived from the movement, Evaluation on the lymphedema using the analysis of body composition

**11-2 Assessment of Efficacy Criteria and method [6], [8], [9], [12], [13], [17], [23], [24], [25], [26], [27]**

1. Shoulder range of motion

The Shoulder ROM (name of equipment) which measures 4 kinds of active range of motion using the universal full-circle manual goniometer is used for measurement.


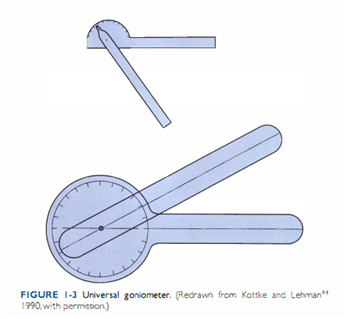


**<Shoulder flexion>**

The movement of abduction and flexion are measured at the coronal, scapular plane and sagittal planes in the neutral anatomical position.


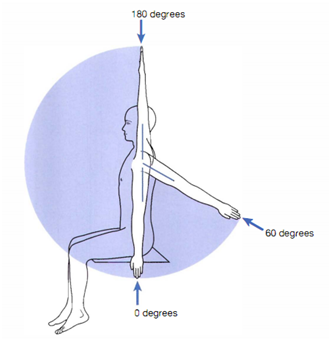


**<Abduction>**


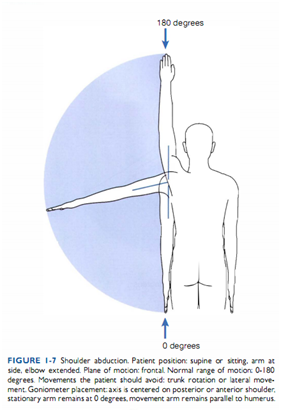


**<External rotation>**

The movement of external rotation is measured with his/her shoulders abducted at 90° degrees, elbows flexed at 90° degree, forearms pronated in the lying and standing position. When measuring in standing position, the patient’s forearms shall be placed to the floor horizontally and his/her palms shall be directed toward the floor.


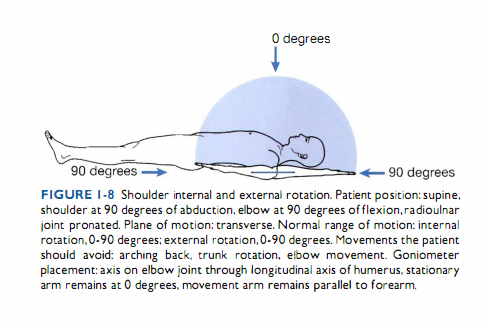


**<Horizontal abduction>**

With his/her shoulders abducted at 90° degree and elbows flexed at 90° degrees, the fully abducted angle shall be measured at the horizontal plane.

(2) Evaluation on the upper limbs’ locomotive capacity (Questionnaire – DASH)

** Refer to the attached

(3) The evaluation on pain derived from the movement

The subject selects the pain level within the diagram designed using VAS (visual analogue scale is the verified assessment tool., 0 means the absence of pain and 100 means the worst pain) stated in the questionnaire, and the examiner records the distance between the starting point and the ending point after measuring it using the ruler at the case report form.

** Refer to the attached

(4) Evaluation on the lymphedema using the analysis of body composition

After analyzing the body composition using Inbody S10(Body composition analyzer), evaluate the degree of arm’s lymphedema which occurred near the operated breast.

**11-3 Assessment of Safety Criteria and method**

(1) Laboratory Tests

Hematologic Tests – WBC, Neutrophil

(2) Evaluation of Adverse Events

Investigators shall educate the subject to report the adverse events voluntarily and occasionally and check the adverse events which occurs in the subject by interviewing or reviewing the report kept in hospital during the visit.

(3) The evaluation on pain derived from the movement

(4) Drainage volume subsequent to the surgery

Drainage volume (ml) unit shall be surveyed.

(5) Expected Adverse Events

In case of the infection which occurs as an adverse events following the use of investigational medical device, we confirmed its degree by checking up the clinical sign and performing the blood test (the white blood cell count (WBC)) scheduled to 7th following the surgery.
In case of the allergic reactions (generalized eruption, respiratory dyspnea, edema, pain, and inflammation), we analyzed the causal relationship with the usage of investigational device by checking up the clinical signs.

**11-4 Timeline of exam and schedule**

The subject will participate in the clinical trial after being approved as the suitable subject who isn’t included in the exclusion/inclusion criteria through the screening test and assigned to the control and the investigational group randomly.

The observation items to be checked during each visit according to the study timeline are as follows.

Clinical trial Schedule

| Items | Treatment and Observation Period | | | | | |
| --- | --- | --- | --- | --- | --- | --- |
| No. of Visits | ^Visit 1^ | ^Visit 21^ | | Visit 3 | Visit 4 | Visit 5 |
|  | Before surgery | Immediately after surgery | 7 days after surgery | 3 month after surgery | 6 months after surgery | 12 months after surgery |
| Visit Window (Day) |  |  | ± 2 | ± 14 | ± 14 | ± 30 |
| Inclusion/ Exclusion Criteria | ○ |  |  |  |  |  |
| Informed Consent | ○ |  |  |  |  |  |
| Basic Information and Medical History | ○ |  |  |  |  |  |
| Hematologic Tests^2^ | ○ |  | ○ |  |  |  |
| Measurement of drainage volume³ |  | ○ | ○ |  |  |  |
| Analysis of body composition | ○ |  |  | ○ | ○ | ○ |
| Investigational medical device prescription |  | ○ |  |  |  |  |
| Measurement in the range of shoulder joint’s motion | ○ |  |  | ○ | ○ | ○ |
| Evaluation on the upper limbs’ locomotive capacity | ○ |  |  | ○ | ○ | ○ |
| Evaluation of pain | ○ |  |  | ○ | ○ | ○ |
| Evaluation of Adverse Events |  |  | ○ | ○ | ○ | ○ |

1) The test for blood and the adverse events scheduled on the visit 2 will be evaluated based on the adverse events discovered on the discharge date of patient or on the 7th day subsequent to the surgery by revisiting the patient.

2) Hematologic Tests - WBC, Neutrophil

The blood test to be performed on the 7th day subsequent to surgery will be conducted only if the adverse events such as the flare onto the surgical site or the fever exists following the surgery. The intention of above-mentioned test (test item: CBC, CRP, and etc.) is to check whether the inflammation factor is increased and the number of white blood cell within 7 days from the surgery.

3) Measurement of drainage volume - Shall be measured at an interval of two days until the 7th day from the surgery date and complete the measurement of it on the discharge date from hospital.

① Visit 1 (Screening visit)

 Written consent signed by subjects

 Investigation on the basic information about the subject, medical history and previous medical history related to shoulder joint

 Preoperative histological diagnosis

 Laboratory Tests were conducted

 Physical function (Shoulder range of motion: Abduction, Horizontal abduction, Flexion, External rotation), Evaluation on the upper limbs’ locomotive capacity, the evaluation on pain derived from the movement

 The analysis of body composition

 Evaluation on the subject eligibility for this clinical trial

② Visit 2(OPD~POD #7)

 The investigational device was applied (The EG, The CG Randomization)

 Histopathological diagnosis and TNM stage

 Postoperative adjunctive therapy (cancer chemotherapy, anti-hormone therapy, target therapy, radiotherapy)

 Postoperative total capacity

 Laboratory Tests were conducted (POD #7)

 Evaluation of Adverse Events

③ Visit 3, 4, 5(POD 3, 6, 12 months)

 Physical function (Shoulder range of motion: Abduction, Horizontal abduction, Flexion, External rotation), Evaluation on the upper limbs’ locomotive capacity, the evaluation on pain derived from the movement

 The analysis of body composition

 Evaluation of Adverse Events

**12. Expected AE and Precautions**

**12-1 General precautions**

(1) This product is sterilized, and its sterilized status must be maintained. Do not use products that have passed the expiration date.

(2) Check the contents before use. And the investigational device shall not be used when the damage of container and package or the presence of moisture is discovered.

(3) The surgeon shall remove the extra liquid of Guardix SG using the aspirator before applying the solution of Guardix SG onto the surgical site.

**12-2 Handling Precautions**

(1) The solution of Guardix SG shall be stored and used at the room temperature.\

(2) The solution of Guardix SG can be used in gel state changed after warming it to body temperature.

(3) Guardix SG shall be sterilized and shall not be used after be re-sterilized.

(4) The remaining solution shall be discarded and shall not be reused since it is for single use.

**12-3 Contraindications**

(1) Contraindicated patients

a. Patients whose surgical site is infected or contaminated.

b. Patients who are hypersensitive to GUARDIX SG

(2) Administration with care

a. The safety and efficacy of GUARDIX SG in its use together with other adhesion prevention products has not been confirmed in animal experiments.

b. It is not recommend to use the solution of Guardix SG during the pregnancy.
It is recommended to plan the pregnancy until the complement of first menstrual period after use of Guardix SG’s solution.

**12-4 Expected adverse events when using the anti-adhesive agent**

Infection: Fever and increase in WBC, Allergic reactions: Systemic rash, dyspnea, and edema, Pain, Inflammation

* Guardix SG (Anti-adhesion) is to prevent the adhesion having a physical barrier in order for the fibroid tissue to infiltrate to the damaged tissue. We recorded the degree of adverse events ranging from mild, moderate, and severe depending on the patient and the state since its degree is diverse regardless of same symptom.

(1) Mild: The degree of adverse events is mild.

(2) Moderate: The degree of adverse events is moderate.

(3) Severe: The degree of adverse events is severe.

**12-5 Expected adverse events during and after the surgery**

Bleeding, infection on wound, necrosis of skin flap, Reduction of shoulder joint’s movable range, annular syndrome on breast, seroma, nerve damage and paresthesia, lymphedema, pain and discomfort in neck, back, and arm, tightening feeling in the skin of surgical site

**12-6 Expected AEs in association with anesthesia are as follows**

Liver toxicity, Kidney toxicity, oliguria, urorrhagia, myoglobinuria, Low blood pressure, high blood pressure, brachycardia, tachycardia, blood pressure change, arrhythmia, abnormal EKG, decreased cardiac output, ventricular extrasystole, supraventricular premature beat, atrial premature contraction, atrioventricular block, bigemina,, Coughing, respiratory disorders, apnea, asthma, hypoxia, laryngospasm, Hyperthermia, hypothermia, and malignant hyperthermia, Anxiety, dizziness, spasmodic movements, agitation, derangement, muscular stiffness, Vomiting, nausea, shivering, and headaches, Allergic reactions such as anaphylactic and anaphylactoid reactions, WBC increase, WBC decrease and temporary blood glucose increase, Muscle aches, feeling of listless, CPK increase

**13. Procedure of drop-out and withdrawal**

When the subject stops to participating in or is dropped out from the clinical trial, the result collected from the discontinued date until the time her discontinuation is handled as a withdrawal will be reviewed during the final evaluation if the result’s item can be used for evaluation.

**13-1 Suspension Criteria**

The sponsor considers continuation or suspension of the clinical trial in a part or all of the institutions according to the protocol if the following events develop.

(1) Investigators can discuss with the sponsor to suspension a part or all of the clinical trial considering the developments observed during the study period, and if necessary, a part or all of the clinical trial process can be discontinued by investigators.

(2) Can suspension a part or all of the clinical trial based on safety or management reasons.

(3) If undesirable developments are observed during the study period, investigators are required to make a request to the IRB to suspension the clinical trial. The clinical trial can be suspension according to the decision from the IRB.

(4) The clinical trial can be suspension if the institution cannot cope with the necessity of modifying the protocol.

(5) The clinical trial can be suspension if cannot accept the modification order from the head of the institution, which was based on IRB opinions.

(6) The clinical trial can be suspension if the institution head is ordered to suspension the clinical trial according to IRB opinions.

(7) The clinical trial can be suspension if the institution seriously or continuously offends the KGCP, protocol, or clinical trial contract.

**13-2. Process of Suspension**

(1) If decides to suspension the clinical trial, his/her intention and reasons of decision must be promptly notified to the institution head in writing.

(2) Upon receiving the suspension notification from the sponsor, the institution head must convey the intention and reasons to the PI and IRB in writing.

(3) Upon receiving the notification of suspension from the sponsor via the institution head, the PI must promptly notify subjects of the meaning of suspension, and guarantee appropriate treatment and follow-up measures.

(4) In the case of suspension, subjects are treated according to the protocol.

(5) If the PI terminates the clinical trial early or temporarily suspension the clinical trial without an agreement with the sponsor, the PI must immediately inform the sponsor and IRB, then submit the details in writing.

(6) If the IRB determines early termination or temporary suspension, the PI must inform the sponsor immediately, and submit details of early termination and temporary suspension in writing.

**13-3. Withdrawal**

Registration means a random assignment (receipt of subject number) of subjects who participated in this clinical trial. Dropped out means a subject who was applied with the investigational medical device at least once, but he/she could not complete the clinical trial process due to any reason.

**13-4. Withdrawal Criteria**

(1) The clinical trial can be withdrawn if the required tests cannot be conducted due to subject conditions or other reasons.

(2) The clinical trial can be withdrawn if subjects show serious AE, adverse device effect (ADE), or serious AE (SAE), which make it difficult to continue with the clinical trial.

(3) The clinical trial can be withdrawn if a serious offence of the protocol develops.

- The clinical trial can be discontinued due to the offence of the inclusion criteria, which can affect the efficacy evaluation.

(4) The clinical trial can be withdrawn if the subjects or legally authorized representatives want discontinuation through withdrawal of the written consent.

(5) The clinical trial can be withdrawn if subjects are not followed up.

(6) The clinical trial can be withdrawn if investigators determine it should be

**13-5. Process of Withdrawal**

(1) Dropout cases must be appropriately managed and treated, and the development must be observed and recorded. The reason of withdrawal must be recorded in the CRF.

(2) All the test results up to the time of withdrawal, the date, reasons, treatments and progress at the time of withdrawal must be recorded in the CRF.

(3) If subjects do not show up for a visit, mail, interview, and phone calls can be used to investigate the reasons and progress, and the results are recorded in the CRF.

(4) The dropout subjects are not replaced with new subjects.

(5) If subjects withdraw in the middle of the clinical trial, they are not required to explain the reason, but investigators must try to confirm the reason upon considering the subject rights.

(6) If there is an outcome of efficacy and safety evaluation (such as AE and concomitant medication) at the time of withdrawal, it must be included in the analysis unless this distorts the validity of the clinical trial.

(7) The result of pregnant subject can be collected only for ethical purpose and the observation on that subject will be continued even after the drop-out.

**13-6 Protocol Violation**

If a violation of protocol is confirmed, investigators must inform the investigator must decide continuation or discontinuation of the clinical trial. The details and reasons of the protocol violation must be recorded in the CRF. Continuation or discontinuation of the clinical trial is determined by the investigators considering the effects of the violation on the efficacy and safety of the investigational device.

**14. Assessment of Efficacy Criteria**

**14-1. Assessment of Efficacy Criteria [6], [8], [9], [12], [13], [17], [23], [24], [25], [26], [27]**

The preoperative and postoperative physical function will be measured.

- Shoulder range of motion (Abduction, Horizontal abduction, Flexion, External rotation)

- Evaluation on the upper limbs’ locomotive capacity

- The evaluation on pain derived from the movement

**14-2. Assessment of Safety Criteria**

Whether the adverse events occur or not and the abnormal findings on laboratory test

**14-3. Assessment of Other Criteria**

The postoperative drainage volume, postoperative therapy (cancer chemotherapy, anti-hormone therapy, target therapy, radiotherapy)

**15. Evaluation Methods and Interpretation Methods**

**15-1 Assessment of Efficacy Methods and Interpretation**

(1) Evaluate using the questionnaire prepared by the researcher and subject

(2) Primary Efficacy Criteria Analysis

The each range of motion’s angle is measured at 4 states of ROM (range of motion) using the universal full-circle manual goniometer on the preoperative day and the 3th month, the 6th month, and the 12th month following the surgery. The measured value is used to analyze the significance at each ROM’s state comparatively by performing the one side and two sample student T-test with the difference and the variance between two groups measured at each period (from preoperative day to the 3th month, the 6th month, and the 12th month following the surgery). When there is over 10 degree differences between two groups (that is the lowest value of confidential interval is over 10), the investigational group is superior to the control. (The over 10 degrees difference of two group is considered to be significant clinically when comparing it).

(3) Secondary Efficacy Criteria Analysis

- Evaluation on the upper limbs’ locomotive capacity (questionnaire – DASH (disability of the arm, shoulder and hand))

The score of DASH questionnaire consist of 30 questions is calculated based on the items filled by the subject.

**DASH disorder/symptom score=[Sum of score filled item]/n-1]25**

* n refers to the number of question filled by the subject and the DASH disorder/symptom score can’t be calculated when more than 3 items weren’t filled.

The calculated scores will be used to analyze the significance comparatively by performing the one side and two sample student T-test with the variances of between two groups measured at each period (from preoperative day to 3, 6, and 12 months following the surgery).

- The evaluation on pain derived from the movement

The subject selects the pain level within the diagram designed using VAS (visual analogue scale is the verified assessment tool., 0 means the absence of pain and 100 means the worst pain) stated in the questionnaire, and the examiner measures the distance between the starting point and the ending point after measuring it using the ruler. The calculated values will be used to analyze the significance comparatively by performing the one side and two sample student T-test with the variances of between two groups measured at each period (from preoperative day to 3, 6, and 12 months following the surgery).

**15-2 Safety Criteria Analysis**

(1) Adverse Events

All the AE that developed during the study period were analyzed. Their onset rate, the onset rate of AE that caused dropout, and the onset rate of SAE were summarized by group. They were coded using WHO-ART, then tested through the Chi-square test or Fisher’s exact test. The onset rates of AE were suggested as: onset rate of all adverse events, and the onset rate associated with the investigational device.

(2) Laboratory Tests

In the case of baseline continuous data from the laboratory test, descriptive statistics quantity (such as mean, SD, median, minimum, and maximum) was calculated, and their normal distribution was tested, then the difference between the groups was compared using One side, Two sample student t-test. In the case of categorical data, their descriptive statistics quantity (frequency and percentage) was suggested, and the difference between the groups was compared using the Chi-square test or Fisher’s exact test. After checking the investigational group’s test value came out on preoperative day and the 7th day following the surgery is different from it of control, we checked whether this difference is included within the normal error range or not.

1) Assessment of Criteria: Adverse events (Visit2, Visit3, Visit4, Visit5) and Hematologic Tests (Visit1, Visit2)

2) Evaluation criteria: The safety of investigational device is considered to be confirmed if the difference between the investigational group’s result value and control’s result value isn’t significant.

**15-3 Statistical Analysis**

(1) Methods: One side, Two sample student t-test, Chi-square test, Fisher’s exact test

(2) Main analysis: ITT analysis

(3) Additional analysis: PP analysis

(4) Statistical power: 80%

* When performing the statistics for effectiveness evaluation, the information of subject who stopped to participate in or was dropped out from clinical trial is excluded from the compliance group (per protocol group) and is dealt as a missing value when the subject is assigned to the analysis group according to assignment table.

(5) General Principals of statistical analyses

The subject data of this study were analyzed through ITT (Intention to Treat) analysis and PP (Per Protocol) analysis.

In the ITT analysis, all the data obtained from the subjects who used the investigational device at least once after they were randomly assigned to the group were used. The above-mentioned subject’s information is excluded from the analysis for primary and secondary the missing value at the primary and secondary is discovered.

In the PP analysis, ITT analysis was data obtained from the subjects who completed the clinical trial according to the protocol were included. And the above-mentioned subject’s safety endpoint shall be included in ITT group. The value of subject which is out of clinical trial’s policy critically and may affect the efficacy evaluation (Exclusion criteria 2. The subject who is diagnosed as a breast cancer, absent on visit 1 and 2(each), assigned to the incorrect group, and experiences the severe adverse events during the clinical trial) will be included in the ITT group analysis with being excluded from the PP group analysis.

The efficacy evaluation was conducted using The efficacy evaluation was conducted using the ITT and PP analysis as a main method, while the PP analysis was used as an auxiliary method when the analysis results were inconsistent. The results of both analyses were compared. Demographical evaluation and safety evaluation were basically analyzed through the ITT analysis.

(6) Demographical Data

After calculating the descriptive statistics (Frequency, standard deviation, minimum value, maximum value, and percentage ratio) for the demographic and medical factors, the Independent t-test and Wilcoxon’s rank sum test shall be applied for continuous data between two groups while the Chi-square test and Fisher’s exact test shall be applied for categorical data between two groups to compare and exam the significance of descriptive statistics

**16. Assessment of Safety Criteria, and Evaluation and Side Effect Report (including adverse events)**

**16-1 Assessment of Safety Methods**

The adverse events confirmed at the physico-chemical exam or reported from the subject are included in the analysis for evaluating the occurrence rate of adverse events which occurred within the investigational group and control and its severity and its type.

(1) Adverse events

All the AE that developed during the study period were analyzed. Their onset rate, the onset rate of AE that caused dropout, and the onset rate of SAE were summarized by group. They were coded using WHO-ART, then tested through the Chi-square test or Fisher’s exact test. The onset rates of AE were suggested as: onset rate of all adverse events, and the onset rate associated with the investigational device.

(2) Laboratory Tests

In the case of baseline continuous data from the laboratory test, descriptive statistics quantity (such as mean, SD, median, minimum, and maximum) was calculated, and their normal distribution was tested, then the difference between the groups was compared using the independent t-test or Wilcoxon’s rank sum test. In the case of categorical data, their descriptive statistics quantity (frequency and percentage) was suggested, and the difference between the groups was compared using the Chi-square test or Fisher’s exact test.

**16-2 Adverse Events Definitions**

(1) Adverse Events, AE

AE is all the unintended syndromes (including signs and lab test results), symptoms, or diseases, which develop in the subjects during the clinical trial period. They are not necessarily associated with the investigational device. Expected daily fluctuations in the test disease or previous clinical manifestations, which are not clinically significant, are not required to be reported.

All AE that developed since the beginning of the clinical trial must be reported. The time of starting the clinical trial is the time of receiving the signed written consent. (Written consent must be obtained before special diagnoses or treatment is started.) All the AEs that developed after the written consent was received must be reported regardless of the administration or association with the investigational device.

(2) Adverse Device Effects, ADE

ADE is the hazardous and unintended reactions caused by the investigational device, whose causal relationship with the investigational device cannot be denied.

(3) Serious AEs · ADEs

One of the following AE or ADE that developed during the clinical trial period due to the investigational device.

1) Death or life-threatening danger

2) Hospitalization or extension of hospitalization

3) Permanent or serious impairment, or deteriorated functions

4) Fetal deformity or anomaly

(4) Unexpected Adverse Device Effects, UADEs

Not considered as significant ADE patterns and hazards according to the information related to the investigational device, as specified in the Investigator’s brochure or attached documents.

**16-3 Adverse Events Report**

The PI and SI(s) are responsible to record all the AE that develop during the clinical trial period. AE must be recorded using medical diagnostic terminologies. If not possible, terminologies of all the symptoms and signs, which were observed by the PI or investigators, or reported by subjects, must be recorded. All the symptoms and signs that developed prior to the administration of the investigational device must be recorded as raw data in the CRF, and those developed after the administration must be recorded in the adverse event section of the CRF regardless of their association with the device.

AE is evaluated by the PI or investigators. The first AE is recorded in the monitoring file of the CRF. Symptoms and signs of AE, measures taken, the onset date and time (if possible), maximal intensity, course (consistent or intermittent), outcomes, and causal relationship with the investigational device are recorded in the CRF.

The AE marked as ‘previously recorded in CRF, or in progress’ is reviewed during the subsequent visits if necessary. When AE is solved, its record must be completed in the CRF. If the frequency and severity of AE increase during the study period, a new record must be started on the AE record file.

In addition to voluntary reports, subjects are asked at every visit questions that do not induce coerced answers such as, “Don’t you feel something different since the last visit or administration?” In the case of non-serious AE, investigators record AE on the basic documents and CRF, and the monitors confirm and review them. SAE is required to be reported to the sponsor within 24 hours after being noticed by the investigators. SAE is also reported to the IRB immediately (within 24 hours) or on the interim report according to the regulation of institutions, whereas, any unexpected ADE must be reported immediately to the sponsor and IRB.

In case of the sponsor-initiated clinical trial, the researcher shall report the occurrence of adverse events to a sponsor and IRB after preparing the report on adverse events when reporting the adverse events. The sponsor shall take a measure for adverse events when necessary after reporting it to IRB and MFDS according to the procedure stated in Appendix 1 form. In case of researcher-initiated clinical trial, only one researcher shall report it to IRB and MFDS directly.

(1) The Investigator must promptly report all the serious and unexpected AE cases (including ADE, serious AE/ADE) to investigators, IRB and MFDS within the period as stated below.

1) Death or life-threatening cases: Within 7 days after the sponsor was notified of or acknowledged the case. Details must be additionally reported within 8 days of the initial report date.

2) Other cases of serious and unexpected SAE must be reported within 15 days of the date the sponsor was notified of or acknowledged the case

(2) The Investigator must additionally and periodically report the safety information of A) until the AE is terminated (such as disappearance and unable to follow-up).

(3) When the Investigator reports the case to the MFDS according to 1, in the ADE reports of the MFDS’ Standards for Clinical Trial for Medical Device, Attached Form 1, ‘3) Safety Report’ must be filled out and attached.

**16-4 Evaluation of the Severity of Adverse events**

Adverse events severity is classified according to its maximal intensity, based on the following criteria.

(1) Mild: Daily life (function) is not affected but there are minimal discomforts. Subjects can easily tolerate it.

(2) Moderate: Daily life (function) is affected significantly.

(3) Severe: Impossible to manage daily life (functions).

**16-4-1. Causal Relationship Between AE and the Investigational Device**

Evaluation of the causal relationship is very important to determine the prompt report. The following items are considered for the causal relationship evaluation.

(1) Exposure: Was the subject exposed to the investigational device?

(2) Time course: Did AE develop according to the time course after applying the investigational device?

(3) Likely: Is the AE development explained better with the application of the investigational device than with baseline diseases or other factors?

(4) Dechallenge: Was the AE relieved or disappeared by discontinuing the use of investigational device?

(5) Rechallenge: Was the AE relieved or disappeared by reapplying the device?

Methods of classifying the evaluation results of the causal relationship with AE (by the WHO).

| Classification | Evaluation Results |
| --- | --- |
| Definitely | Valid time course is shown with the application of the investigational device.  Cannot be explained with diseases or other causes.  When discontinued, symptoms react.  When rechallenged, symptoms react. |
| Probable | Valid time course is shown with the application of the investigational device.  Difficult to explain with diseases or other causes.  When dechallenged, symptoms react.  Rechallenge is not required |
| Possible | Valid time course is shown with the application of the investigational device.  Can be explained with diseases or other causes.  When dechallenged, reactions can be mild or unclear. |
| Unlikely | Valid time course may be minimal with the application of the investigational device.  Can be explained with diseases or other causes.  When dechallenged, reactions can be mild or unclear. |
| Unrelated | No time course with the application of the investigational device.  Clearly explained with diseases or other causes.  When dechallenged, no reactions. |
| Unclassified | Unable to evaluate due to insufficient or contradictory information.  Data cannot be supplemented or confirmed. |

The causal relationship between AE and investigational device (or other causes such as baseline disease and combined treatment) is determined based on the following factors:

◆ Known functions of the investigational device

◆ Functions previously observed in the investigational device or the same series device as the investigational device

◆ Reactions that were frequently reported in association with the device similar to the investigational device (e. g.; erythema and blood diseases)

**16-5 AE Follow-Up**

The PI or investigators must follow up with the subjects with AE until their symptoms are relieved, clinical values are recovered to the reference level, or the observed changes are satisfactorily explained. AE safety report must be submitted upon request from the sponsor.

**16-6 SAE**

(1) Definition of SAE

SAE means cases of death, life-threatening events, hospitalization or extension of hospitalization, continuous or critical disability/incapacitating, or congenital deformity or impairment of the offspring of subjects. Death, non-life-threatening events, and cases not requiring hospitalization can be regarded as SAE when they harm the patients or subjects, or when medical or surgical measures are required to prevent the results listed in the definition section above.

1) Definition of "Life-Threatening"

If the development of AE has the possibility resulting in an immediate death of the subject, the AE is life-threatening. However, an AE, which can induce death if the AE becomes more serious, is not included in this category.

2) Definition of "Disability/Incapacitating"

If a patient has an AE that causes substantial or continuous difficulties hindering normal life, the patient is suffering from AE with temporary or continuous disabilities.

3) Definition of "Hospitalization"

AE requiring hospitalization thought to be serious. Preplanned surgery or hospitalization, which was arranged for baseline symptoms with mild severity or frequency prior to the clinical trial, is not classified as AE.

If undesirable symptoms develop during the planned process, those must be reported as serious or non-serious AE according to the classification criteria. These usually involve staying in a ward or emergency room for at least one night because the subject could not be properly treated in the clinical doctor’s office or outpatient clinic. Cases on the border line of hospitalization are regarded as SAE.

4) Definition of "Routine Clinical Procedure"

A clinical procedure that can develop during the clinical trial period but not inhibiting the normal procedure of the protocol

5) Others

Cases of life-threatening, death, or critical risks not requiring hospitalization, which require internal or surgical intervention for preventing the above-mentioned developments, must be addressed with medical or scientific measures. These cases are also regarded as SAE.

(2) SAE Report

1) The investigator must promptly report all the serious and unexpected AE cases (including ADE, serious AE/ADE) to investigators, IRB and MFDS within the period as stated below.

(A) Death or life-threatening cases: Within 7 days after the sponsor was notified of or acknowledged the case. Details must be additionally reported within 8 days of the initial report date.

(B) Other cases of serious and unexpected SAE must be reported within 15 days of the date the sponsor was notified of or acknowledged the case

2) The investigator must additionally and periodically report the safety information of 1) until the AE is terminated (such as disappearance and unable to follow-up).

3) When the investigator reports the case to the MFDS according to 1, in the ADE reports of the MFDS’ Standards for Clinical Trial for Medical Device, Attached Form 1, ‘3) Safety Report’ must be filled out and attached.

**16-7 Report about the safety**

(1) The PI must immediately report all the SAE (except the cases waived in the protocol or clinical study data file) to the sponsor within the period stated in the protocol using the Enforcement Regulations of the Medical Device Act, Attached Form 35. To protect the privacy of subjects, an ID code is used instead of the subject name, resident registration number, and address. The PI must follow any additional guidelines of AE.

(2) The PI must report AE and abnormal laboratory test values, which are listed in the protocol, to the sponsor according to the period and report methods described in the protocol.

(3) When reporting cases of death to the sponsor and IRB, the PI must attach additional documents such as an autopsy report (if applicable) and death certificate.

◆ Investigators’ Responsibilities

(1) The PI must consistently evaluate the safety of the investigational device.

(2) The PI must promptly report any safety information of the investigational device, which can affect the subjects, the continuity of the clinical trial, or the decisions of the IRB, to investigators and MFDS.

**16-8 ADE**

(1) Definition of ADE

Not considered as significant ADE patterns and hazards according to the information related to the investigational device, as specified in the Investigator brochure or attached documents.

(2) Report on the adverse events after using the investigational device

1) The investigator must report all the serious or unexpected ADEs within the period described below to investigators and IRB (Only when the PI have not reported to the IRB, or when to revise the already reported information).

2) The investigator must report additional information of ADE, which was reported according to 1, until the ADE is terminated (disappearance of the ADE or inability of follow-up investigation).

3) When the PI reports ADE using a prompt report form (Attached Form 35) to the MFDS, the PI’s report, which was prepared according to 7 K 1), must be attached.

**16-9 Pregnancy**

Subjects who became aware of their pregnancy during and after termination of the clinical trial must inform investigators whether they became pregnant during the study period or within 30 days after the last administration of the investigational device. All pregnant subjects must immediately inform the sponsor, and fill out the SAE report form within 24 hours to report to the monitor. The initial pregnancy report must be attached. When SAE on pregnancy is accepted, the investigator sends a pregnancy report form to investigators for their report of additional pregnancy data. All the pregnant subjects are followed up during the pregnancy period. the investigator must send additional forms to investigators for their report on the health status of mothers and babies. All the cases of abortion including artificial abortion must also be reported.

**17. Other Items for Safe and Scientific Clinical Trial**

(1) Case Report Form, CRF

The CRF is a published or electronically prepared written document of each subject, which is to be transferred to the sponsor. (CRF attached)

(2) PI CV

(CV attached)

(3) Use and control of the investigational device

The investigational device is controlled by the person who was designated by the head of the clinical trial institution. The investigational device must be used and stored as described in its manual, and indicated with “For clinical trial.” The investigational device manager receives and returns the medical device, controls inventory, and maintains records.

(4) Supply and control of the investigational device

Prior to the clinical trial, investigators explain the details of the clinical trial, investigational device, effects and AE, and safety; then, subjects voluntarily sign the written consent form. When subjects or legally authorized representatives cannot read the written documents, impartial witnesses can participate in the consent process. In this case, the consent form, information letter and other written documents must be read and explained to subjects or legally authorized representatives to have them orally consent, and if possible, to have them sign and date the consent form. At the same time, impartial witnesses must confirm that the consent form, information letter, and other written documents were explained to the subjects or representatives, and they understood the details, then the consent was conducted based on the subjects’ or legally authorized representatives’ free will. (Information letter and written consent form attached)

**18. Agreement on Compensation**

The patient who participated in this clinical trial is subject to this term of compensation for victim.

**18-1 Reason of compensation**

The physical damage which occurs to the subject due to the deleterious and unintended reaction subsequent to the usage of investigational device even when the concerned staff complies with the guideline of investigational device’s usage will be compensated under the term of compensation and related laws if the case of victim falls under the following category.

(1) In case of the temporary pain or a mild damage to be cured easily which the investigational site decided the treatment is necessary (The compensation range is limited to the treatment cost).

(2) Hospitalization or extension of hospitalization

(3) Permanent or serious impairment, or deteriorated functions

(4) Fetal deformity or anomaly

(5) Death or life-threatening danger

**18-2 Requirements for compensation**

The subject will be compensated when the case of victim satisfies the below-mentioned requirements.

(1) The physical damage occurs after using the investigational device.

(2) The investigator complies with the guideline stated on the protocol of clinical trial approved by the MFDS.

(3) The reason of subject’s damage isn’t derived from the mistake or a negligence of investigator’s duty.

(4) The subject complies with the guidelines related to the clinical trial fully.

(5) The subject took a measure to minimize the occurrence of damage derived from physical damage.

**18-3 The reason for exemption of compensation**The following cases are excluded from the compensation range if the case satisfied the previously stated requirements (Article 18-2).

(1) The damage which occurs due to the ineffectiveness of investigational device to the indication

(2) The damage derived from the carelessness of subject in use

**18-4 Criteria for compensation**

(1) When there is a monetary amount or the type of medical treatment agreed between two parties as a reward for a damage derived from the expected adverse events, the subject will be compensated according to that term.

(2) Otherwise, the compensation cost will be decided in consideration of the degree of physical damage, characteristics, duration period, the similar case, and etc. by the method of compensation agreed between two parties.

(3) When the consent on all terms concerning the compensation isn’t reached, the subject will be compensated according to the verdict in the court or the decision which is commensurate with.

**18-5 Procedure of compensation**

The subject damaged physically even when she follows the guidelines of clinical trial shall request the required medical treatment to the investigator or investigative institution. That the subject didn’t notify the other party of the objection after the subject is treated means two parties agree with the term of compensation stated on the content of statement notified to two parties. If the subject notifies the other party of objection, the other party recommends the plural objective experts who appraise the criteria for the inclusion of subject to the compensation and the compensation criteria to the subject then subject shall nominate one person among nominee within 3 days from the recommendation date. (If the subject didn’t nominate one person, the deputy of subject shall select one person randomly.

**18-6 Scope of Application**

(1) This compensation agreement is generally applied to all subjects who participate in the clinical trial unless another agreement is applied to the subjects and the sponsor.

(2) Any compensation agreement between the subjects and a third party, which was not approved by the sponsor, has no legal or contractual effects on the sponsor.

We will try to ensure that the participants of this clinical trial are not disadvantaged from the aforementioned items. We hereby pledge to abide by the agreement.

. . 2015.

Asan Medical Center, Breast endocrine surgery, Professor Son Byung Ho (seal)

**19. Subject Care After Clinical Trial**

Dropped out participants or subjects who do not show any reactions to this clinical trial must be advised to try appropriate alternative treatments, and the subjects who do not show treatment effects after this clinical trial must be referred to other treatment methods. Subjects with SAE or deterioration in diseases during or after the clinical trial period can be treated according to the directions from Principal Investigators or investigators, and compensated according to the victim compensation agreement.

(1) Principals

1) Provides treatment and compensation for any physical damages (including death) that occurred to subjects.

2) If the damage developed due to the investigational device, must treatment and compensate the subjects.

3) Subjects that are severely damaged who may have chronic impairments are treatment and compensated.

4) If the damage developed as a side effect of the investigational device, or in the side-effect treatment process, the victim is treatment and compensated if the damage was directly resulted from the investigational device.

(2) Victims are not treatment or compensated in the following cases

1) The damage derived from adverse events which occurred due to usage of other medical device, not the investigational device

2) Failure to provide subjects with expected effects or benefits from the use of the investigational device

3) Any damages that developed due to the breach of the agreed protocol (or due to the failure to comply with the directions of clinical investigators

4) Any damages that arisen from subjects’ carelessness

(3) Compensation Evaluation Criteria

1) Compensation must be determined considering the property, severity, and continuity of the damage. The amount should be reasonable in comparison with precedent cases set by the Korean court.

2) When subjects are in disagreement in regard to the compensation from advice from professionals who are accepted by both parties must be sought.

(4) Compensation Procedure

1) Request for compensation – Contact listed in the information letter

2) Review of compensation – Details are investigated and compared

3) Review of compensation by advisory doctor and insurance company - The amount of compensation is determined. (When the subject participates in the clinical trial after buying the insurance)

4) Payment of compensation - to subjects or representatives

We will try to ensure that the participants of this clinical trial are not disadvantaged from the aforementioned items. We hereby pledge to abide by the agreement

**20. Subject Action to Take for Subject Safety**

**20-1 Clinical Trial Institution**

Clinical trial institutions must be appropriately equipped with a clinical laboratory, and have professional staff members to cope with any emergencies that may occur during the clinical trial period. When the clinical trial is discontinued or completed for the protection of subject at an early stage or temporarily, the principal of investigative site shall submit the detailed explanatory statement on it after reporting it to the principal of MFDS.

**20-2 IRB**

Institutional Review Board (IRB) must be organized according to Korean regulations/practices. The IRB must protect the rights, safety, and welfare of subjects, and the eligibility of subjects in the vulnerable environment must be closely examined.

The IRB can discontinue a part or all of the clinical trial if subjects’ consent were not appropriately obtained; the clinical trial is not conducted according to the protocol; or SAE/AE develops. When the subject receives the monetary reward in return for the participation in the clinical trial, the IRB shall review the amount and method of reward and whether the monetary reward affects the subject adversely. If the subject received the monetary reward, IRB shall review the amount of monetary reward is enough in view of the participation period and the degree of participation and whether the monetary reward is provided on the condition of that the subject shall participate in the clinical trial until the end of it. In addition, IRB shall check whether the compensation plan for the subject who didn’t complete the clinical trial exists.

**20-3 Investigators**

Investigators include PI, investigators, and clinical trial coordinators. Investigators must conduct the clinical trial in compliance with the protocol that was agreed with the sponsor, and approved by the IRB and MFDS.

When enrolling the subject, the concerned staff shall get the voluntary consent from the subject and make all subjects understand the content of clinical trial after explaining about the content of it in detail before starting the clinical trial. During or after the clinical trial, the investigator shall take a measure in order for the subject to be treated medically when the adverse events occurs including the abnormal result of laboratory test which has a clinical significance and notifies the subject of the necessity of medical treatment for the occurred disease discovered by the investigator when necessary. The investigator has a responsibility for the compensation of subject and etc., shall supervise the conduction of clinical trial for proceeding it according to the procedure stated on the protocol of clinical trial, and counteract to resolve the problem related to the subject actively. (The CV of principal investigator is attached.)

**20-4 Protocol Approval**

The protocol and related documents must be submitted to the IRB according to Korean regulations before the clinical trial starts. The sponsor and investigator must inform each other in writing of the satisfaction of ethical and legal requirements prior to subject registration. Contract is made upon obtaining approval from the institution. (Clinical trial contract attached)

**20-5 Confidentiality**

All the subject names must be kept confidential, and recorded using the numbers assigned during the clinical trial period. The number must be confirmed at every evaluation of the subject. Subjects are informed when their clinical trial data are saved in computers, and kept confidential. The signed written consent is kept by the investigators. Signing on the protocol means that investigators agreed to duly obtain written consent from the subjects who participate in the clinical trial, and to be put under inspection upon request. Investigators prepare a list of subject numbers and names for convenience. The subject written consent and list are stored for 3 years.

**20-6 Recording and the Use of Clinical Trial Outcomes**

All the data collected from this clinical trial must be recorded by investigators in the CRF that was provided by the investigator. Investigators must keep a copy of the CRF.

The CRF must be filled out clearly and legibly using a black ballpoint pen (Pencil and fountain pen are not allowed.). Missing or omitted data must be explained with reasons by investigators.

When the records of CRF are corrected, the original record must be recognizable, and signed by the corrector. (CRF attached)

**20-7 Others**

The protocol was prepared based on the Declaration of Helsinki, and considering the rights and welfare of subjects. The PI or investigators must explain the purpose and all the possibilities of this study. Only subjects who voluntarily signed the written consent can be targeted. (Appendix. Declaration of Helsinki)

Investigators and researchers must be well acquainted with the protocol, and the PI must be able to cope with unexpected AE. The PI reports AE, educates researchers, and conducts necessary measures while proceeding with the clinical trial according to the ‘Standards for Management of Clinical Trial for Medical Devices’

**21. Reference**

1. Kootstra JJ, Weebers J, Rietman JS, Vries JV, Baas PC, Geertzen JH, Hoekstra HJ (2010) A longitudinal comparison of arm morbidity in stage I-II breast cancer patients treated with sentinel lymph node biopsy, sentinel lymph node biopsy followed by completion lymph node dissection, or axillary lymph node dissection. Ann Surg Oncol 17:2384-2394

2. Hayes SC, Rye S, Battistutta D, DiSipio T, Newman B (2010) Upper-body morbidity following breast cancer treatment is common, may persist longer-term and adversely influences quality of life. Health Qual Life Outcomes 8:92

3. Kwan W, Jackson J, Weir LM, Dingee C, McGregor G, Olivotto IA (2002) Chronic arm morbidity after curative breast cancer treatment: prevalence and impact on quality of life. J Clin Oncol 20:4242–4248

4. Yang EJ, Park WB, Seo KS, Kim SW, Heo CY, Lim JY (2010) Longitudinal change of treatment-related upper limb dysfunction and its impact on late dysfunction in breast cancer survivors: a prospective cohort study. J Surg Oncol 101:84–91

5. Cheville AL, Tchou J (2007) Barriers to rehabilitation following surgery for primary breast cancer. J Surg Oncol 95:409–418

6. Stubblefield MD, Custodio CM (2006) Upper-extremity pain disorders in breast cancer. Arch Phys Med Rehabil 87:S96–S99 (quiz S100–S101)

7. Karki A, Simonen R, Malkia E, Selfe J (2005) Impairments, activity limitations and participation restrictions 6 and 12 months after breast cancer operation. J Rehabil Med 37:180–188

8. Lee SA, Kang JY, Kim YD, An AR, Kim SW, Kim YS, Lim JY (2010) Effects of a scapula-oriented shoulder exercise programme on upper limb dysfunction in breast cancer survivors: a randomized controlled pilot trial. Clin Rehabil 24:600–613

9. Shamley D, Srinaganathan R, Oskrochi R, Lascurain-Aguirrebena I, Sugden E (2009) Three-dimensional scapulothoracic motion following treatment for breast cancer. Breast Cancer Res Treat 118:315–322

10. Caban ME, Freeman JL, Zhang DD, Jansen C, Ostir G, Hatch SS, Goodwin JS (2006) The relationship between depressive symptoms and shoulder mobility among older women: assessment at one year after breast cancer diagnosis. Clin Rehabil 20:513–522

11. Agrawal A, Ayantunde AA, Cheung KL (2006) Concepts of seroma formation and prevention in breast cancer surgery. ANZ J Surg 76:1088–1095

12. Lotze MT, Duncan MA, Gerber LH, Woltering EA, Rosenberg SA (1981) Early versus delayed shoulder motion following axillary dissection: a randomized prospective study. Ann Surg 193:288–295

13. Shamley DR, Barker K, Simonite V, Beardshaw A (2005) Delayed versus immediate exercises following surgery for breast cancer: a systematic review. Breast Cancer Res Treat 90:263–271

14. Mais V, Bracco GL, Litta P, Gargiulo T, Melis GB (2006) Reduction of postoperative adhesions with an auto-crosslinked hyaluronan gel in gynaecological laparoscopic surgery: a blinded, controlled, randomized, multicentre study. Hum Reprod 21:1248–1254

15. Kim JH, Lee JH, Yoon JH, Chang JH, Bae JH, Kim KS (2007) Antiadhesive effect of the mixed solution of sodium hyaluronate and sodium carboxymethylcellulose after endoscopic sinus surgery. Am J Rhinol 21:95–99

16. Park JS, Cha SJ, Kim BG, Choi YS, Kwon GY, Kang H, An SS (2011) An assessment of the effects of a hyaluronan-based solution on reduction of postsurgical adhesion formation in rats: a comparative study of hyaluronan-based solution and two film barriers. J Surg Res 168:49–55

17. Yang EJ, Kang E, Jang JY, Kim D, Yom CK, Lim JY, Kim SW (2012) Effect of a mixed solution of sodium hyaluronate and carboxymethyl cellulose on upper limb dysfunction after total mastectomy: a double-blind, randomized clinical trial. Breast Cancer Res Treat. 136:187-194.

18. Falk K, Björquist P, Strömqvist M, Holmdahl L (2001) Reduction of experimental adhesion formation by inhibition of plasminogen activator inhibitor type 1. Br J Surg. 88:286-289.

19. Burns JW, Colt MJ, Burgees LS, Skinner KC (1997) Preclinical evaluation of Seprafilm bioresorbable membrane. Eur J Surg Suppl. 577:40-48.

20. Kwon SW, Lim SH, Lee YW, Lee YG, Chu BY, Lee JH, Lee YM (2006) Anti-adhesive Effect of Poloxamer/Alginate/CaCl2 Mixture in the Rat Model. J Korean Surg Soc. 71:280-287

21. Kong CG, In Y, Cho HM, Suhl KH (2011) The effects of applying adhesion prevention gel on the range of motion and pain after TKA. Knee. 18:104-107

22. Park JH, Jeong JJ, Kang SW, Nam KH, Hang Chang HS, Chung WY, Park CS (2006) The Efficacy and Safety of Guardix SG^Ⓡ^ in Patients Who Are Undergoing Thyroid Surgery: A Randomized, Prospective, Double-blinded Study. Korean Journal of Endocrine Surgery 9:127-132

23. de Rezende LF, Franco RL, de Rezende MF, Beletti PO, Morais SS, Gurgel MS (2006) Two exercise schemes in postoperative breast cancer: comparison of effects on shoulder movement and lymphatic disturbance. Tumori. 92:55-61

24. Box RC, Reul-Hirche HM, Bullock-Saxton JE, Furnival CM (2002) Shoulder movement after breast cancer surgery: results of a randomised controlled study of postoperative physiotherapy. Breast Cancer Res Treat. 75:35-50.

25. M. M. F., Oliveira; M. S. C., Gurgel; M. S., Miranda; M. A., Okubo; L. F. A., Feijó; G. A., Souza (2009) Efficacy of shoulder exercises on locoregional complications in women undergoing radiotherapy for breast cancer: clinical trial. Brazilian Journal of Physical Therapy 13:136-143

26. Lim JY, Lee HY, Song JH, Kang JW, Lee JY (2005) Evaluation of the Reliability, Construct Validity, and Responsiveness of the Korean Version of the DASH. The Joumal of the Korean Society for Surgery of the Hand. 10:192-198

27. D. Gould et al. (2001) Visual Analogue Scale(VAS). 706

28. Chow SC, Shao J, Wang H (2008) Sample size calculations in clinical research (second edition). Taylor & Francis Group, LLC.
